# Critical Parameters to Improve Pancreatic Cancer Treatment Using Magnetic Hyperthermia: Field Conditions, Immune Response, and Particle Biodistribution

**DOI:** 10.1021/acsami.1c02338

**Published:** 2021-03-12

**Authors:** Lilianne Beola, Valeria Grazú, Yilian Fernández-Afonso, Raluca M. Fratila, Marcelo de las Heras, Jesús M. de la Fuente, Lucía Gutiérrez, Laura Asín

**Affiliations:** † Instituto de Nanociencia y Materiales de Aragón (INMA), CSIC—Universidad de Zaragoza, 50018 Zaragoza, Spain; ‡ Department of Analytical Chemistry, Universidad de Zaragoza, 50018 Zaragoza, Spain; § Centro de Investigación Biomédica en Red de Bioingeniería, Biomateriales y Nanomedicina (CIBER-BBN), 50018 Zaragoza, Spain; ∥ Department of Animal Pathology, Universidad de Zaragoza, 50009 Zaragoza, Spain

**Keywords:** iron oxide magnetic nanoparticles, magnetic hyperthermia, intratumor administration, biodistribution, immunological effect, pancreatic cancer

## Abstract

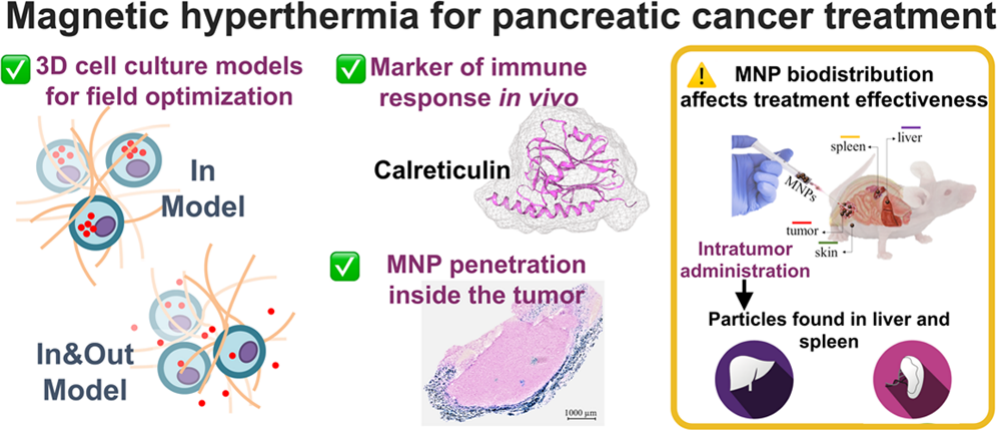

Magnetic hyperthermia (MH) was used to treat a murine model of pancreatic cancer. This type of cancer is generally characterized by the presence of dense stroma that acts as a barrier for chemotherapeutic treatments. Several alternating magnetic field (AMF) conditions were evaluated using three-dimensional (3D) cell culture models loaded with magnetic nanoparticles (MNPs) to determine which conditions were producing a strong effect on the cell viability. Once the optimal AMF conditions were selected, *in vivo* experiments were carried out using similar frequency and field amplitude parameters. A marker of the immune response activation, calreticulin (CALR), was evaluated in cells from a xenograft tumor model after the MH treatment. Moreover, the distribution of nanoparticles within the tumor tissue was assessed by histological analysis of tumor sections, observing that the exposure to the alternating magnetic field resulted in the migration of particles toward the inner parts of the tumor. Finally, a relationship between an inadequate body biodistribution of the particles after their intratumoral injection and a significant decrease in the effectiveness of the MH treatment was found. Animals in which most of the particles remained in the tumor area after injection showed higher reductions in the tumor volume growth in comparison with those animals in which part of the particles were found also in the liver and spleen. Therefore, our results point out several factors that should be considered to improve the treatment effectiveness of pancreatic cancer by magnetic hyperthermia.

## Introduction

Pancreatic ductal adenocarcinoma (PDAC), the most frequent type of pancreatic cancer, is characterized by the generalized presence of extremely dense stroma. This stroma is responsible for the increased rigidity of these tumors^1^ and acts as a barrier against chemotherapeutic drugs, not only decreasing their effectiveness but also impairing infiltration of antitumor immune cells.^2^ This, among other factors, is one of the reasons for the bad prognosis of this type of cancer, with average 5-year-survival rates of 10%. Therefore, new therapeutic strategies for the treatment of this disease, which trigger both the selective death of tumor cells and tackle the disruption of stromal components, are urgently needed. In fact, several clinical trials have recently focused on the tumor–stroma interactions as an alternative therapeutic approach for this type of tumors.^3^

Over the past few decades, nanotechnology has been extensively exploited in the quest for new and advanced tools for diagnosis and therapy of diseases. Different types of nanomaterials have been proposed as biosensors, as contrast agents for different imaging techniques, or as nanocarriers able to improve the efficacy, long-term stability, and biodistribution of drugs.^4^ In the frame of pancreatic cancer, several nanoparticle-based strategies have been investigated for the development of new diagnostic and therapeutic platforms, with some of them showing potential for the modulation of the PDAC microenvironment. Most of the proposed strategies have focused on the design of complex drug delivery systems, combining in the same nanoplatform several therapeutic agents to promote either tumor cell death or stroma disruption.^5^

Our treatment proposal is a single nanomaterial, iron oxide magnetic nanoparticles, with a dual role: having the capacity of changing the stroma permeability and provoking tumor cell death when an alternating magnetic field (AMF) is remotely applied.^6^ Our previous work using three-dimensional (3D) cell culture models composed of collagen, which is the main component of the extracellular matrix, showed that the heat produced by iron oxide magnetic nanoparticles (MNPs) was able to produce cell death, triggered by intracellular nanoscale hotspots, and also improve the permeability of the collagen, acting as an extracellular matrix disruptor allowing the penetration of the nanoparticles toward the inner part of the 3D structure.^6^ As mentioned before, the capacity of disrupting the extracellular matrix is especially relevant in the frame of PDAC, in which the stroma can account for up to 80% of the tumor volume, acting as a biological barrier against anti-PDAC treatments and therefore reducing their effectiveness.

The proposed therapeutic approach for achieving the above-mentioned effects is nanoparticle-based magnetic hyperthermia (MH). In this kind of therapeutic treatment, magnetic nanoparticles are able to convert energy absorbed from an external alternating magnetic field (AMF) into local heat. This treatment had been proposed either as a standalone method or in combination with other treatments.^7^ In fact, the European Medical Agency approved MH treatment for clinical practice, after the success of trials for glioblastoma (Berlin, Charité Hospital, 2003–2005). This treatment was also approved as an adjuvant therapy for recurrent glioblastoma in combination with radiotherapy in 2012.^8^ Currently, the company MagForce is undertaking clinical trials in the United States to validate its use also for prostate cancer.^9^ Despite these advances, researchers working in the field of magnetic hyperthermia for cancer treatment still face several challenges and practical problems such as the difficulty in achieving enough magnetic material in the tumors after intravenous administration or the heterogeneous distribution of the particles in the whole tumor volume even after intratumoral injection.^10^ In addition, there are still many knowledge gaps in the frame of *in vivo* MH applications, such as the cytotoxicity mechanisms triggered directly by the heat or the immune response activation stimulated by the treatment.

In the specific case of pancreatic tumors, some *in vitro* and *in vivo* studies had addressed the use of MH to treat this challenging tumor.^11−15^ The novelty of this work is that we focused on three different aspects of PDAC treatment using magnetic hyperthermia that we thought should be considered with regard to clinical practice: (i) the selection of the AMF conditions using 3D *in vitro* models, which mimic more properly the cellular environment than two-dimensional (2D) ones; (ii) the evaluation of the possible immune response to the treatment; and (iii) the impact of undesired leakage of MNPs from the tumor on the treatment effectiveness. The reasons for focusing on these three aspects are more thoroughly described below.

First, it was shown that the aggregation^16^ or alignment^17^ of particles within cells has a strong impact on their heating properties. This means that, although it is possible to easily characterize the heating properties of an MNP suspension in water, the real heating properties inside the cells will still be a difficult value to estimate. This problem becomes even more complex considering that it is hard to estimate the local MNP concentration inside the cells and that, moreover, the changes in the heating properties with the particle concentration are not linear.^18^ Therefore, it is difficult to predict how efficient will be the heat produced by the MNPs *in vivo*. Thus, using *in vitro* models, and in particular 3D cell cultures, is a good approach to produce a relatively high number of replicas to perform MH tests, reducing the number of animals needed. In this work, we used 3D cell models to test several AMF conditions and evaluate which one was producing a stronger effect on the cells before going to *in vivo* experiments.

Second, the antitumor immune response is a key factor in the effectiveness of cancer treatments but had been largely ignored in the analysis of the response to MH treatments. Gaining knowledge on how MH treatment could affect the immune system response would allow taking advantage of natural defense mechanisms against tumors. One of the events that could be evaluated to assess the response of the immune system to a treatment is the presence of damage-associated molecular patterns (DAMPs) in the treated cells.^19^ One of the several DAMPs that could be analyzed is the expression in the cell surface of calreticulin (CALR), which occurs early in the course of immunogenic cell death (ICD).^20^ In general, DAMPs bind to pattern recognition receptors (PRRs) in the immune cells, leading to the activation of both innate and adaptive immune responses. Stimulating the immune system with MH treatment would lead to very important advantages, such as generating a systemic antitumor reaction that could act in metastatic situations.^21^ This would be especially relevant for PDAC, which metastasizes frequently to the liver.^22^ Furthermore, activation of the immune responses would also trigger an immune memory effect that could prevent patients from relapses.^23^

Third, it was reported that one of the main problems when administering MNPs intravenously to perform any treatment was that the amount of material that reaches the desired location is very low (less than 1%).^24^ This is the reason why the majority of the studies of hyperthermia *in vivo* used the direct injection of the magnetic material at the tumor site as the main route of administration,^25^ as this procedure ensured that a high concentration of the MNPs remained in the desired site. However, to date, little attention has been paid to the number of particles that remain in the tumor after intratumoral administration and the possible impact that a particle leakage to other organs may have on the treatment effectiveness.

In this work, these important parameters related to the antitumor effectiveness of MH for pancreatic cancer treatment both *in vitro* and *in vivo* were evaluated. First, several field conditions were tested in 3D cell culture models, using a pancreatic tumor cell line (MIA PaCa-2) and collagen, to select the field amplitude and frequency that resulted in the strongest response to treatment. Our previous works, studying the increase of MNP internalization due to AMF exposure and also the death pathway triggered depending on the MNP dose,^6,26^ were performed using a macrophage cell line, known for its high MNP internalization capability. Therefore, to evaluate the feasibility of using MH for PDAC treatment, a pancreatic tumor cell line was needed to optimize the AMF conditions. Then, the MH treatment was tested in a heterotopic xenograft mouse model after intratumoral administration of the magnetic nanoparticles. The expression of calreticulin, a marker of immune response activation, was measured in tumor cells after *in vivo* application of MH treatment, paying special attention to differences between cells that had or had not internalized particles. The tumor volumes were measured repeatedly over a month until the mice were sacrificed to evaluate the *in vivo* effectiveness of the therapy. In addition, the penetration of the MNPs toward the inner parts of the tumors after AMF exposure was measured by histological assessment of tumor sections. As differences in the tumor growth were found between animals that received the whole treatment (MNPs + AMF application), the impact of MNP leakage to other organs, in particular, the liver and the spleen, on treatment effectiveness was evaluated through particle quantification from tissue alternating current (AC) magnetic susceptibility measurements. The evaluation of these key factors showed that they have a strong impact on the final antitumor effectiveness of MH and thus should be considered while searching for optimal treatment schemes for pancreatic cancer using this therapeutic approach.

## Results and Discussion

Magnetic nanoparticles from the same synthetic batch as in our previous works were used in this study.^6,26^ Briefly, oleic acid-coated 11.3 ± 0.2 nm spherical iron oxide nanoparticles (Figure S1 from the Supporting Information) were synthesized by thermal decomposition and stored in hexane. The magnetic cores were then coated with poly(maleic anhydride-alt-1-octadecene) (PMAO) modified with a fluorophore (TAMRA, carboxytetramethylrhodamine), to allow stabilizing them in water and tracking them *in vitro* and *in vivo*. Finally, the PMAO-coated particles were functionalized with glucose to produce the final material used in this work. This functionalization step was carried out to prevent aggregation in complex biological media and improve the particle uptake by the cells.^27^ The specific absorption rate (SAR) value for these particles was 104 W/g Fe, (measured at *H* = 20 kA/m and *f* = 829 kHz, using [Fe] = 1 mg/mL). Their ζ-potential in water was −8.3 ± 1.0 mV, and the hydrodynamic size, from dynamic light-scattering measurements, was 85 ± 7 nm (Figure S1 from the Supporting Information).

### Development of Two 3D Cell Culture Models

**Figure 1:**
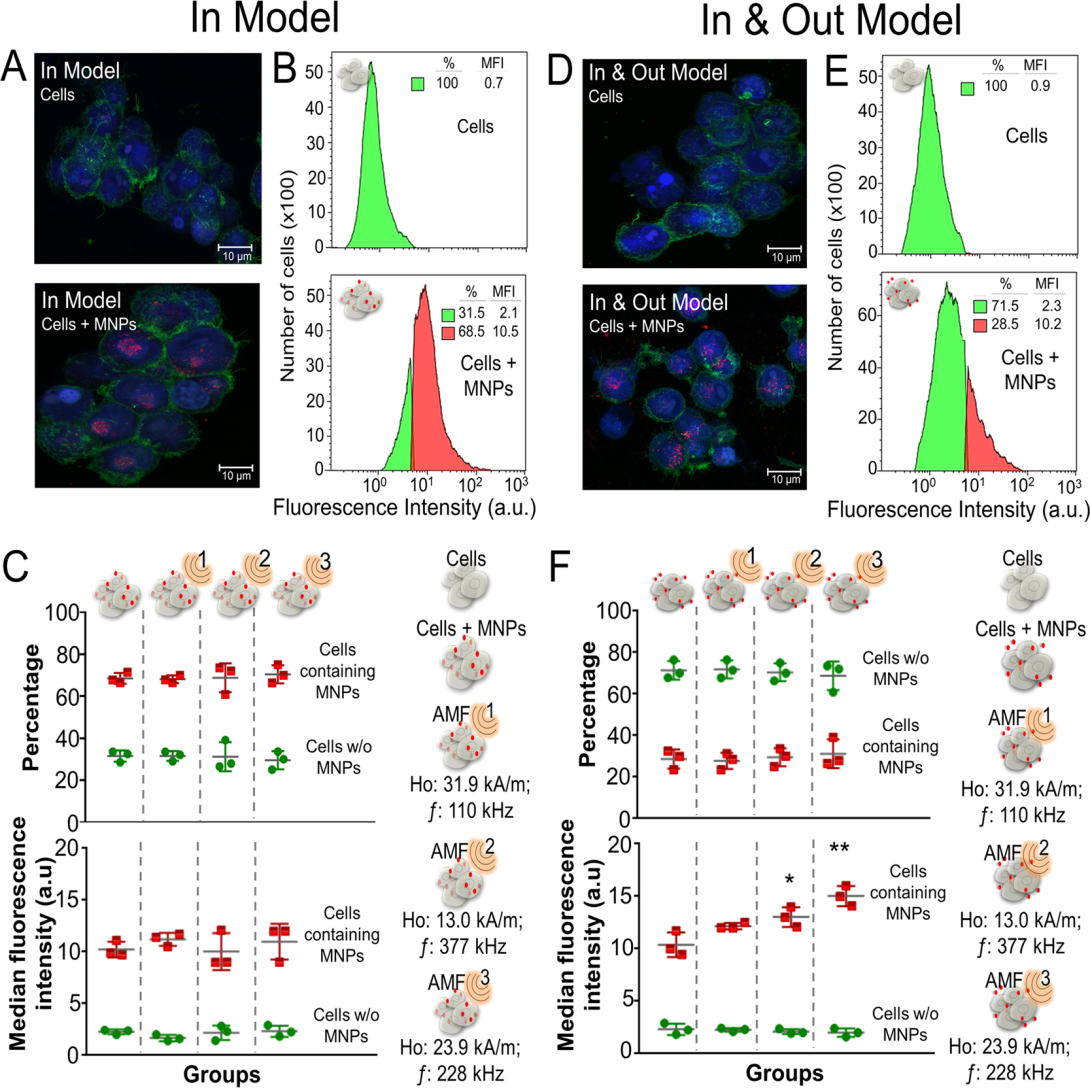
3D cell culture model characterization: In Model (left) and In&Out Model (right). (A, D) Confocal microscopy images. Red, MNPs (TAMRA); blue, 4′,6-diamidino-2-phenylindole (DAPI; nucleus); green: Alexa Fluor 488 Phalloidin. Scale bar is 10 μm. (B, E) Flow cytometry analysis of nanoparticle uptake. A representative histogram from three independent experiments is shown in the figure; in green is the cell population with no particles and in red is the cell population that contained internalized particles. (C, F) MNP uptake before and after exposure to different AMF conditions (AMF 1: 110 kHz; 31.9 kA/m. AMF 2: 377 kHz; 13 kA/m. AMF 3: 228 kHz; 23.9 kA/m) measured as a percentage of cells with MNPs and the changes in the MFI obtained from flow cytometry data. Statistical differences were determined using a two-way analysis of variance (ANOVA) followed by Sidak’s multiple comparisons test (**p < 0.01; *p < 0.05; *p* > 0.05 no significance).

As in our previous works,^6,26^ we prepared two different 3D cell culture models mimicking different *in vivo* scenarios of MNP internalization. We called the first model “In Model”. In this model, cells seeded in 24-well culture plates were incubated with the MNPs for 24 h; after this time, the cells were washed to remove the unbound MNPs and then used to form the 3D collagen gel. This procedure resulted in a high percentage of MNP-loaded cells, as shown by flow cytometry (FC) (68.5 ± 2.6%, Figure 1B). In this model, as expected, a high number of internalized particles was achieved and no extracellular MNPs were found in the collagen matrix, as was verified with fluorescence microscopy (Figure 1A). The second model, named “In&Out Model”, consisted of a collagen gel, where the cells were previously embedded and that was subsequently incubated with MNPs for 24 h. This second model was chosen as it mimics better the real situation that may occur in the tissues with particles being located both inside and outside the cells. In this model, a lower number of MNP-loaded cells was found (28.5 ± 4.5%, Figure 1E) in comparison with the other model. In addition, MNPs were still found in the collagen matrix outside the cells (Figure 1D). Although the percentage of cells that internalized MNPs in both cases was very different (with the number of cells with internalized MNPs being much higher in the In Model than in the In&Out one), the average amount of MNPs internalized per cell was very similar. This could be inferred by the values of median fluorescence intensity (MFI) of both positive populations (10.2 ± 0.8 and 10.4 ± 1.2 au for In Model and In&Out Model, respectively) pointing out to a similar internalization capability of the cells regardless of whether they were preloaded with MNPs in a 2D culture or not before being entrapped in the collagen matrix. We had previously shown that the procedure used to load the 3D gel with MNPs in the In&Out Model yielded a heterogeneous distribution of the particles within its structure, where most of the particles were located in the outer areas of the 3D structure.^6^ The difficulty of the MNPs to reach the inner parts of the 3D structure in this model was the reason for the lower number of cells that were able to internalize the MNPs, when compared to the In Model.

In previous experiments made with macrophages, we also observed that extracellular MNPs in the In&Out Model diffuse with time to the inner part of the structure.^6^ This migration was faster if the 3D cell cultures were exposed to an AMF, probably because the heat generated by the particles disrupted the collagen fibers increasing the permeability of the 3D collagen structure. In this work, we studied the influence that the exposure to an AMF had on MNP internalization in tumor cells in both models. Three different combinations of magnetic field amplitude (*H*) and frequency (*f*) were used (AMF 1: 110 kHz and 31.9 kA/m; AMF 2: 377 kHz and 13 kA/m; and AMF 3: 228 kHz and 23.9 kA/m). These conditions were selected, within the technical limitations of our device, to maintain the biological limit considered safe in the literature.^28,29^ In all cases, the AMF exposure was 30 min.

As expected, in the In Model, both the percentage of MNP-loaded cells and the MFI remained the same before and after the AMF application due to the absence of MNPs located extracellularly (Figure 1C). In contrast, in the In&Out Model, while the percentage of MNP-loaded cells did not change significantly before and after the AMF application, the average amount of MNPs internalized per cell increased after the MH treatment (Figure 1F). These results indicated that probably longer times are needed to achieve a complete internalization of the particles by the cells that are located in the inner part of the 3D structure.

Interestingly, the increase in the number of MNPs internalized per cell in the In&Out Model after the AMF treatment was significantly different depending on the AMF conditions. In particular, the highest increase in MNP internalization was observed for AMF 3 conditions (MFI = 15.0 ± 0.9 au). This value was the highest of all of the conditions tested at the different AMF conditions (Figure 1C,F).

Probably, the variations in the AMF conditions produced a different heating profile both on the collagen matrix and on the plasma membrane of MNP-loaded cells that had an effect on the diffusion of the MNPs within the 3D structure and its internalization. Several approaches had been described to measure temperatures locally, although all of them needed a significant alteration of the MNPs,^30,31^ and therefore, the results may not be a precise reflection of the real nanoscale local temperatures reached during the AMF exposure. SAR measurements of water suspensions of these particles were also not a good characterization alternative to estimate such values, as, among other limitations, it is very difficult to know the local MNP concentration in the 3D cell culture, a key parameter affecting the interparticle dipolar interactions and thus their heating efficiency.^32^ Further studies using theoretical simulations^18^ would be required to completely understand the heat being released by the particles under the AMF conditions that were applied to further optimize the treatment.

In these experiments, the use of the In&Out Model was especially interesting as it helped highlight that the cell internalization of MNPs located in the extracellular environment was enhanced by the MH treatment. This observation could have an important effect in clinical settings, as in most *in vivo* studies the MNPs are injected intratumorally,^11,33,34^ where a remarkable percentage of MNPs remains in the extracellular matrix. In such a case, a positive effect of the repetition of AMF exposure on consecutive days to enhance the effect of MH treatment may occur.^35,36^ Additionally, an increase of the extracellular matrix permeability after the AMF exposure could be of great help not only because of the MNP internalization enhancement but also because it could be used to allow the infiltration of antitumor drugs or immune system cells toward the inner areas of the tumor that would contribute to improving the final antitumor effectiveness.^37^

### Use of 3D Cell Culture Models to Select the AMF Conditions with Better Effectiveness

After studying the MNP internalization, we examined the cell viability after MH treatment at the three conditions of magnetic field detailed above. For that purpose, the cell viability of the different treatments was tested by flow cytometry using propidium iodide (PI), which enters the cell as a result of plasmatic membrane disruption, and Annexin V, which binds phosphatidyl serine (PS), a phospholipid that translocates to the outer leaflet of the plasmatic membrane at the beginning of the apoptosis process.

**Figure 2:**
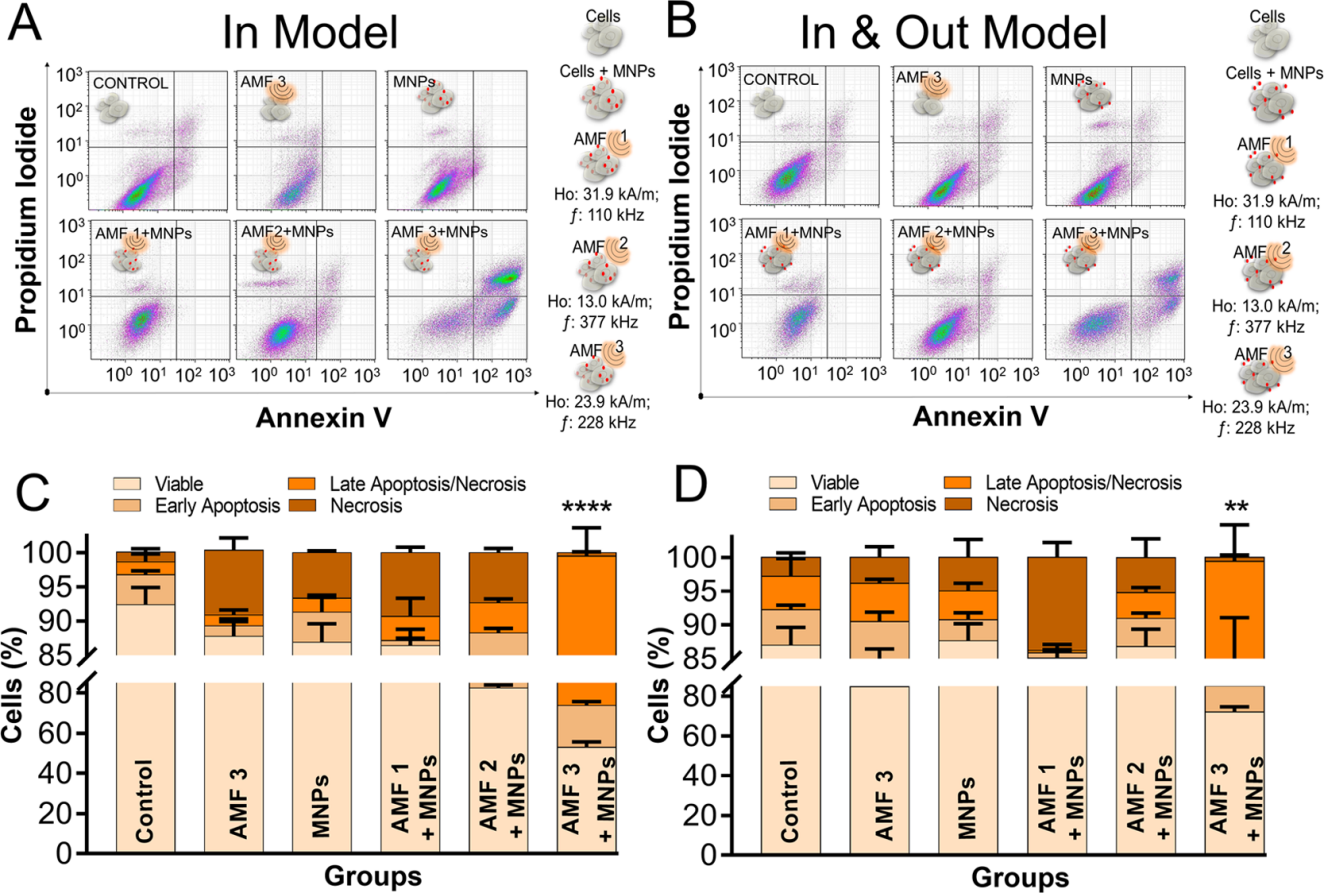
Cell death induction (Annexin V/PI staining) 24 h after a single administration of magnetic hyperthermia treatment using different AMF conditions. In Model (left) and In&Out Model (right). AMF 1: 110 kHz, 31.9 kA/m. AMF 2: 377 kHz, 13 kA/m. AMF 3: 228 kHz, 23.9 kA/m. (A, B) Selected density plots representative of three independent experiments. Control experiments using AMF 1 and AMF 2 are shown in Figure S2 of the Supporting Information. (C, D) Summarized result data resulting from three independent experiments shown as mean ± SD. Significant differences with respect to the percentage of apoptotic cells between the control group and the treated groups were analyzed using a two-way ANOVA followed by Dunnet’s multiple comparisons test (****p < 0.0001; **p < 0.01; *p* > 0.05 no significance).

Figures 2 and S2 from the Supporting Information show the results of the cell viability studies 24 h after the MH treatment for both 3D models, In Model and In&Out Model. It was observed that, for both models, all of the controls presented cell viability values above 80%, confirming that the AMF and the MNPs *per se* did not produce any cytotoxic effect. When analyzing the results of cell viability after the complete treatment using three different AMF conditions, it was observed that, in both models, when using AMF 1 and AMF 2 conditions the cell viability was similar to the control groups. These results indicated that the heat released by the MNPs in these conditions was not enough to produce detectable cellular damage in MIA PaCa-2 cells at least 24 h after the treatment. However, results were different for the AMF 3 condition, where the cell viability decreased in both models (72.0 ± 2.6% for the In Model and 52.9 ± 2.8% for the In&Out Model). In these conditions, cells positive for Annexin V (undergoing early apoptosis) and cells positive for both PI and Annexin V (undergoing apoptosis or necrosis) were detected. The differences in the percentages of dead cells observed for the two models under exposure to AMF 3 were probably due to the different percentages of MNP-loaded cells. In the case of the In Model, the percentage of cells that internalized MNPs was much higher than for the In&Out Model, as previously shown in Figure 1, and as a result, the overall cell viability decreased in a bigger proportion. Having these two 3D cell culture models allowed us to determine the importance of increasing the number of cells that internalized particles to achieve better treatment effectiveness.

The type of cell death triggered by MH treatment was the same in both models, observing some cells in an early apoptosis stage and the other population in a late necrosis/apoptosis stage. Having the same death route in both 3D models agreed with the fact that the average number of MNPs per cell, as determined from the mean fluorescence intensity in Figure 1, was similar in both of them at the beginning of AMF application.

Furthermore, these results agreed with the observations for MNP internalization in the previous section, pointing to the fact that these field amplitude and frequency conditions were the ones in which our system released more heat. These results were also in agreement with our previous work comparing both 3D models but using a nontumoral model cell line.^6^ As the *f* × *H*_0_ value from AMF 3 was slightly above the biological limit of 5 × 10^9^ A/ms^28,29^ (5.4 × 10^9^ A/ms), for the *in vivo* experiments, the closest AMF conditions to AMF 3 available in our device (*f* = 196 kHz; *H* = 26 kA/m) were chosen.

### Detection of Markers of Immunogenic Cell Death after MH Treatment

The effect of MH treatment in a murine model was then studied using the AMF conditions similar to those of AMF 3, optimized in the *in vitro* experiments (AMF_in vivo_: 196 kHz; 26 kA/m). A nude mice strain was selected for the subcutaneous implantation of MIA PaCa-2 cells, which led to the development of a xenograft pancreatic tumor in the right flank of the animals. It is important to bear in mind that these athymic animals lack the adaptive immune response orchestrated by the thymus, presenting a reduced number of T cells, which may lead to an underestimation of the real effectiveness of the treatment.

**Scheme 1:**
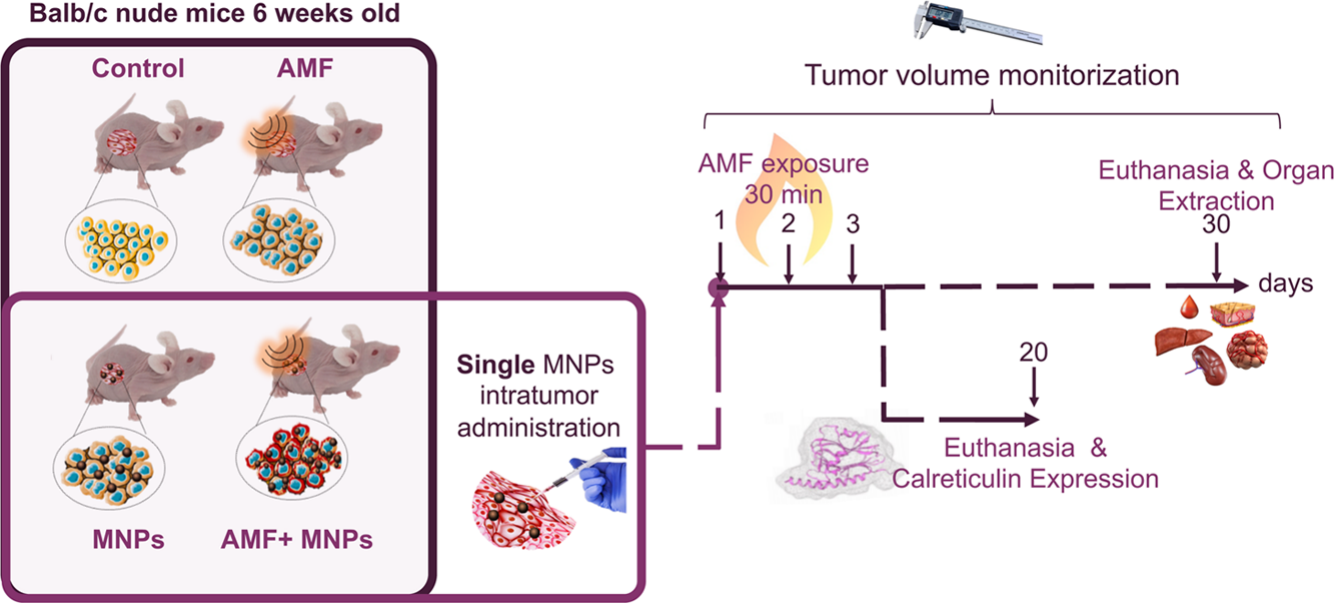
Schematic Representation of the Different Groups Used in the *In Vivo* Experiments and the Timeline of the Experiment Including the AMF Exposure and the Moments in Which the Animals Were Sacrificed for the Different Experiments Control, animals without any treatment; AMF, animals exposed to the AMF but without the MNP injection; MNPs, animals that received the MNP injection but were not exposed to the AMF; and AMF + MNPs, animals that received the MNP injection and were then exposed to the AMF.

Approximately 14 days after the MIA PaCa-2 cells’ injection, the tumors were small (around 100–200 mm^3^), but detectable and appropriate, for MNP injection (0.15 mg Fe/tumor). Animals were randomly distributed in four groups, four mice in each control group and eight in the one receiving the complete treatment. The different experimental groups were as follows: (i) control animals without any treatment (control group); (ii) animals exposed to the AMF but without the MNP injection (AMF); (iii) animals that received the MNP injection but were not exposed to the AMF (MNPs); and (iv) animals that received the MNP injection and were then exposed to the AMF (AMF + MNPs). The groups receiving MH were exposed to the AMF on the same day of the MNP injection and on the next 2 consecutive days (see Scheme 1). In all cases, the evolution of the tumor volume and the animals’ weight were followed twice a week for 4 weeks after treatment, with the exception of the animals in which the CALR expression was studied, which were monitored just for 2 weeks.

At around 20 days after the MNP administration, we assessed the expression of a marker of immunogenic cell death (ICD) during *in vivo* MH treatment to know whether the cells that die during treatment could trigger an immune response that would help in the tumor treatment.

The generation of some damage-associated molecular patterns (DAMPs) is related to immunogenic cell death activation.^38,39^ The expression of these molecules in cells responding to a death stimulus leads to a robust immunostimulatory effect since they bind to pattern recognition receptors in the immune cells. Calreticulin (CALR) proteins, normally located in cellular storage compartments, are exposed on the outer side of the plasmatic membrane in the beginning of the ICD, before the translocation of the phosphatidyl serine typical of apoptosis.^20^ CALR is exposed as a consequence of the activation of caspase 8 and other molecules involved in apoptosis. When CALR is recognized by a lipoprotein receptor (low-density lipoprotein receptor LRP1 or CD91), an important phagocytic signal is triggered, stimulating antigen-presenting cells like the dendritic cells. CALR exposure can be detected by flow cytometry using the corresponding specific antibody.

**Figure 3:**
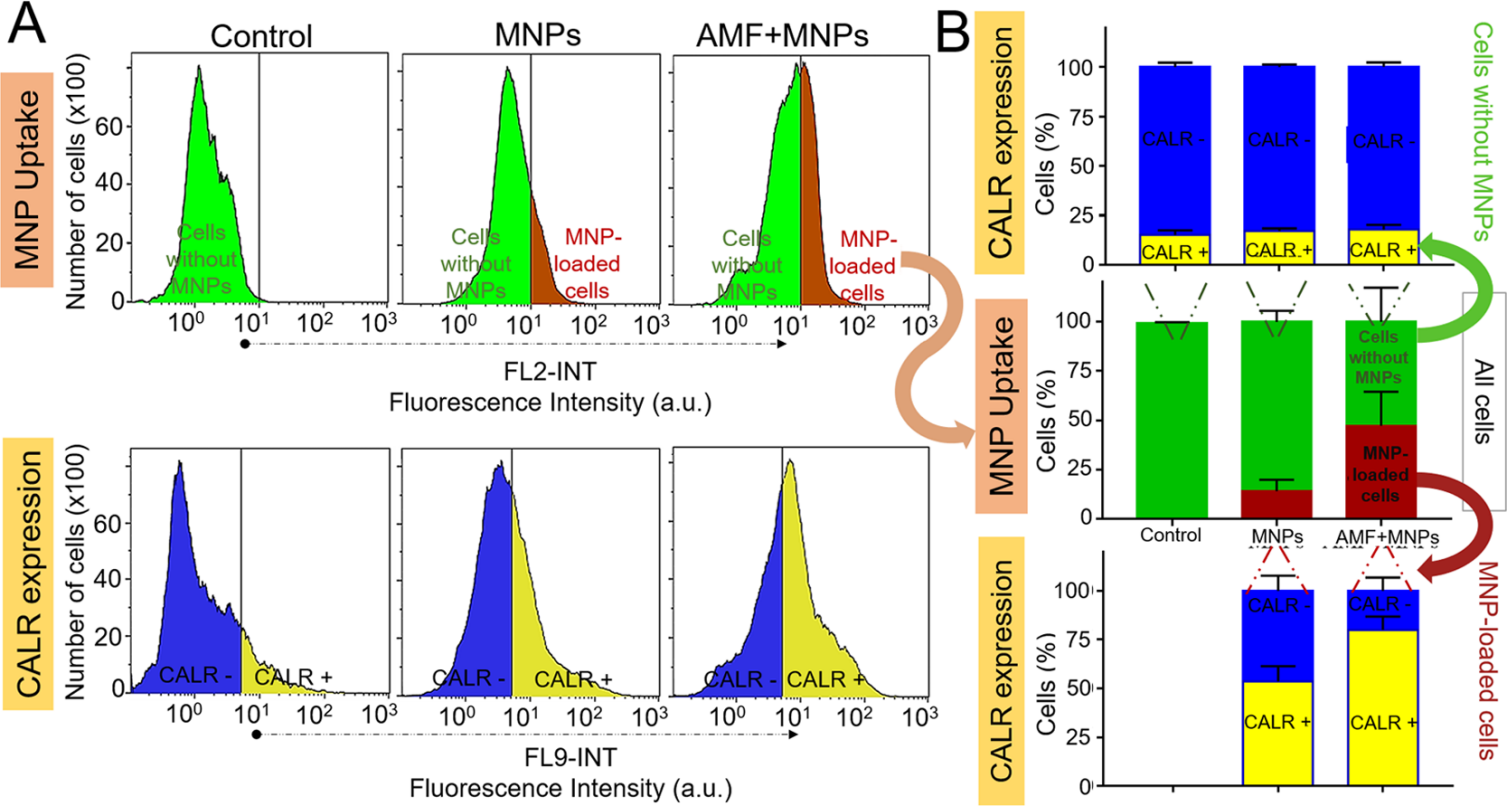
Magnetic nanoparticle uptake and calreticulin expression in animals from the control group, the group that received the MNP intratumor administration (MNPs), and the one that received the complete treatment (AMF + MNPs). (A) Flow cytometry histograms showing MNP internalization (top) and CALR expression (bottom) in cells obtained by digesting the whole tumor after the MH treatment. (B) Summarized data from flow cytometry experiments showing the percentage of cells that express CALR in the membrane in the cells without MNPs (top) and in the MNP-loaded cells (bottom). *n* = 2.

Figure 3 shows the results of CALR expression of three groups of mice: control, MNPs, and AMF + MNPs sacrificed at around 20 days after the beginning of the study. After euthanizing the animals, tumors were extracted, and a cell dissociation protocol was followed to obtain a homogeneous cell suspension formed by both tumor cells and extracellular tumor matrix-infiltrating cells. Although it was known that in the case of these tumors most of the cells were tumor cells, still a small percentage of other cells may be present and as such formed part of the analysis.^40−42^ The complete suspension was then analyzed by flow cytometry determining two parameters: MNP internalization and expression of CALR proteins in the cells obtained after digesting the tumor mass. Results indicated that in the AMF + MNP group the percentage of cells that internalized MNPs was three times higher (∼47%) compared to those animals that received the MNP injection but were not exposed to the AMF (MNP group: ∼14%) (Figure 3). Although this increase in the number of cells that internalized MNPs after the AMF exposure was not observed in the *in vitro* experiments, probably, this could be related to the repetitive consecutive AMF cycles applied and the longer time after the AMF exposure during which MNP internalization was carried out in the animal study compared with the *in vitro* one.

Concerning the CALR expression, it was observed that in the MNP group there was a portion of cells that expressed a higher amount of CALR in their plasma membrane (∼55%; MFI = 9.7 ± 0.1 au) compared to the cells without MNPs (∼15%; MFI = 9.4 ± 0.1 au), which indicated that the internalized MNPs were causing some stress to the cells even in the case of not being exposed to the AMF. Interestingly, the expression of this protein increased considerably for the complete treatment (AMF + MNP group: ∼80%; MFI = 10.6 ± 0.4 au).

To go one step further, these results were analyzed more in detail by comparing the percentage of cells expressing CALR in the two distinguishable populations from each treatment, the cells with and without MNP internalization (Figure 3). In the MNP-treated group, when analyzing only MNP-loaded cells, the percentage of cells positive for CALR expression was 54%, indicating some degree of immunostimulatory effect associated with the presence of MNPs. Even though no clear toxicity of these particles was observed *in vitro* at shorter times (Figure 2), recent works had highlighted the potential use of iron-based nanomaterials for cancer treatment as ferroptosis^43^ activators at longer times once the particles are degraded and release iron atoms.^44,45^ In fact, it was recently shown that the oxidative stress induced by the Fenton reaction, generated by the free iron ions, led to the exposure of calreticulin on tumor cells,^46^ results that support our findings here. The percentage of cells positive for CALR expression from the MNP-loaded cell population of the AMF + MNP treatment was significantly higher (80%) than that of the MNP treatment, indicating that a stronger activation of the immune system could have been triggered after the MH treatment. In contrast, when analyzing the cell population with no internalized MNPs from the three groups (control, MNPs, and AMF + MNPs), the expression of the CALR levels was similar in all of the samples analyzed, indicating that no bystander effect was occurring, at least in terms of the expression of this molecule (Figure 3).

In summary, our results indicated that tumor cells were affected by the presence of internalized particles and that the magnetic hyperthermia treatment enhanced the expression of the analyzed DAMP, indicating that the potential activation of immunogenic cell death routes was triggered by this treatment. These results are in agreement with a recent publication, using also iron oxides, that showed that after heating up tumors with MNPs, in this case with light radiation, there was an immune response, observing the activation of dendritic cells and T cells in *in vivo* experiments, resulting in the inhibition of metastatic tumors.^47^ Indeed, very little work has been performed on the study of the immunomodulatory effect of MH treatment,^21,23,48^ and none of the few reported studies used iron oxide spherical magnetic nanoparticles as thermal sources, as is the case in this work. Some recent work had also evaluated the activation of the immune system in the tumor environment related to the heat produced by photothermal therapy with gold nanoparticles,^49^ and other works had presented the activation of immune responses using magnetic nanoparticles but not exposed to the AMF.^50^ Thus, the emerging field of the use of magnetic nanoparticles to trigger an immune response in the frame of cancer treatment needs further studies to identify all of the relevant factors in the immune system response generated both by the MNPs and by the MH treatment and their impact on therapeutic response. Stimulating the adaptive immune response would open a window of opportunity for the treatment of metastatic tumors, as the generation of a systemic antitumor reaction in the primary tumor could have an effect on secondary tumors and also trigger an immune memory effect,^51^ which could prevent patients from relapsing.

### Observation of Tumor Growth Inhibition after MH Treatment

**Figure 4:**
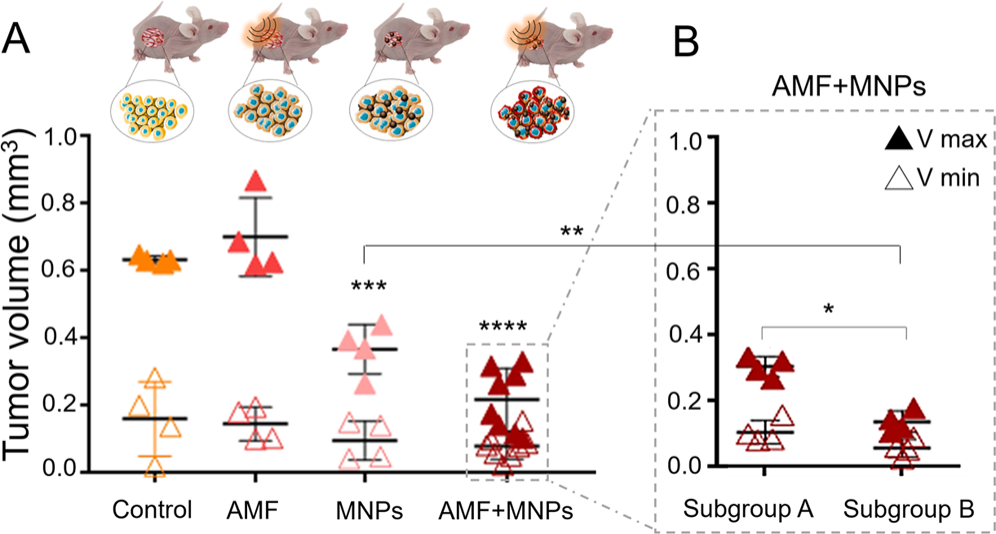
(A) Tumor evolution represented as the starting volume (*V*_min_) and maximum volume reached during the experiment (*V*_max_). (B) Same data as in panel A for the AMF + MNP group but divided into two subgroups with different behaviors: subgroup A, with a lower treatment effectiveness; and subgroup B, with a better response to the treatment. Significant differences with respect to the control were analyzed using a two-way ANOVA followed by Sidak’s multiple comparisons test (****p < 0.0001; ***p < 0.001; **p < 0.01; *p < 0.05; *p* > 0.05 no significance). In cases where more than one group generated significant differences with respect to the control, the means between those groups were also compared. This figure was produced using images from the Servier Medical Art PPT image bank.

To evaluate the MH treatment effectiveness *in vivo*, the evolution of the tumor volume along time for the different groups was followed for approximately 30 days (Figure 4A,B). In general, this is the main parameter that has been used in previous works over the past few decades assessing the effectiveness of the treatment.^25^ Previous *in vivo* studies evaluating the use of MH for cancer treatment generally had reported the inhibition of tumor growth, and there are only scarce works where a complete regression of the tumor occurred.^52−55^

In our work, as expected, the control and the AMF group showed the highest tumor growth rates, very similar in both cases. The MNP group, which received the particle injection, did not reach tumor volumes as high as the other two control groups (control and AMF groups). Finally, the AMF + MNP group, where animals received the complete treatment consisting of three cycles of MH, showed a heterogeneous antitumor effect, which could be divided into two different subgroups (Figure 4B). One of these subgroups showed a lower antitumor effect (subgroup A), while the other subgroup of animals (subgroup B) showed a higher inhibition of tumor growth. The differences in tumor growth behavior between these two subgroups were statistically different.

The reduction in tumor growth in the group treated just with the MNPs in comparison with the control and AMF groups indicated an interesting potential therapeutic effect just from the stress that could be triggered due to the internalization or degradation of magnetic particles, as hypothesized from the results of the CALR expression enhancement in cells containing MNPs (Figure 3). Although stronger effects on tumor growth were observed in the group that received the complete treatment (MNPs + AMF), the differences between animals belonging to the complete treatment group were striking and needed further analysis. Therefore, to evaluate the reasons for these differences, further histopathological analyses and magnetic measurements to shed light on differences in MNP biodistribution after MNP injection were performed.

### Enhancement of MNP Internalization in the Tumor after MH Treatment

**Figure 5:**
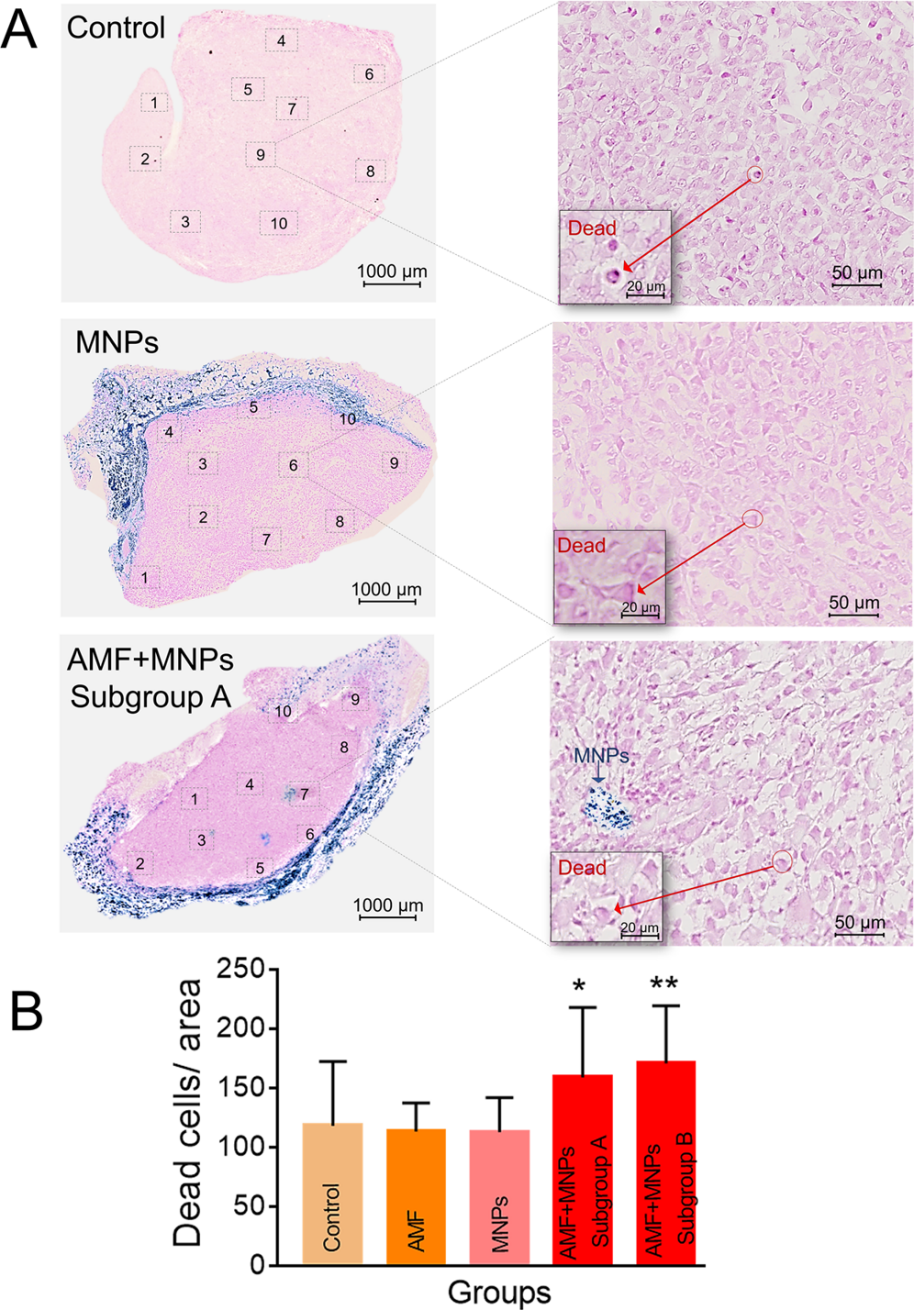
Histological analysis of tumor sections. (A) Representative tumor sections after Perls Prussian blue staining. The numbers represent the 10 random areas used for dead cell quantification in each section. (B) Summarized analysis of the dead cells per area in the different groups analyzed. The data are represented as mean ± SD. The statistical differences with respect to the control group were determined using a one-way ANOVA (**p < 0.01; *p < 0.05; *p* > 0.05 no significance).

Histological assessment of tumor sections from the different groups provided further information regarding the effect of the treatment. The nanoparticle distribution in the tissue was observed through specific iron staining with Perls Prussian blue. In both groups containing nanoparticles (MNPs and AFM + MNPs), the particles were mainly located in the outer areas of the tumor (Figure 5A). This nonhomogeneous particle distribution within the tumor had also been previously reported in other studies using intratumoral administration.^56,57^ Some factors had already been proposed as the cause of the variation of spatial distribution of the nanoparticles in the tumor, such as the flow rate injection,^58^ the time between the injection and the MH treatment, or the exposure or not to the AMF.^59^ This nonhomogeneous distribution of MNPs could be one of the reasons why the complete inhibition of tumor growth was not observed in our case, as the hotspots generated by the particles may not have any impact on the inner areas of the tumor.^60^

In our work, we quantified the dead cells in each tumor section based on morphological changes observed in histological sections (Figures 5 and S3 of the Supporting Information) to obtain more information about the effects caused by MH treatment. Ten different zones of the same area per tissue section were selected for the analysis. No significant differences in the number of dead cells per area were observed between the three control groups: mice treated with PBS (control), mice exposed to the AMF field (AMF), or mice treated with the MNPs but not exposed to the AMF (MNPs) (Figure 5B). In contrast, significant differences were found for the group that received the complete MH treatment (AMF + MNPs) (Figure 5B). These differences were higher in the subgroup of mice where a clear tumor growth inhibition was observed (*p* = 0.0014 for subgroup B). In contrast, a smaller effect was observed in the other subgroup of mice (*p* = 0.0187 for subgroup A). These results agreed with the effect observed in the evolution of the tumor volume found for these two subgroups.

Interestingly, in all of the mice exposed to an AMF after the MNP injection, penetration of the particles toward the inner parts of the tumor was observed (Figure 5). This is the same effect previously reported in our studies with 3D cell culture models^6^ and in previous *in vivo* works.^59,60^ The reason for this penetration is probably the temperature increase triggered by the MH treatment, which can have an important effect on the extracellular matrix structure, facilitating the penetration of MNPs toward the inner areas of the tumor. As mentioned before, these results were especially relevant in the frame of pancreatic ductal adenocarcinoma, given its high content of stroma that acts as a barrier against chemotherapeutic agents.^61^ It could be envisaged that MH treatment could act as a tool able to increase the permeability of the stroma, having potential synergistic effects on the treatment of pancreatic cancer with chemotherapy. Nevertheless, further experiments are still needed to clarify the cause of the differences in effectiveness of MH treatment between the two subgroups that received the complete treatment (subgroup A and subgroup B).

### Hindering of MH Effectiveness by Leakage of MNPs to the Liver and the Spleen

To analyze the possible causes of these differences between the groups of animals that received the complete treatment, we performed an analysis of MNP concentration in the tumor tissues together with the analysis of their biodistribution among other organs once the animals were sacrificed.

Few studies have analyzed the biodistribution of nanoparticles toward the internal organs when using intratumor administration.^52,56^ One of the main difficulties is that, given the relatively high amount of endogenous iron in the liver and the spleen, conventional elemental analysis techniques are not specific enough to detect variations in small amounts of iron coming from the particles. For this reason, AC magnetic susceptibility measurements were selected, as this technique is able to distinguish between the endogenous iron and the iron originating from the MNPs, allowing their detection with high sensitivity and specificity.^62^

**Figure 6:**
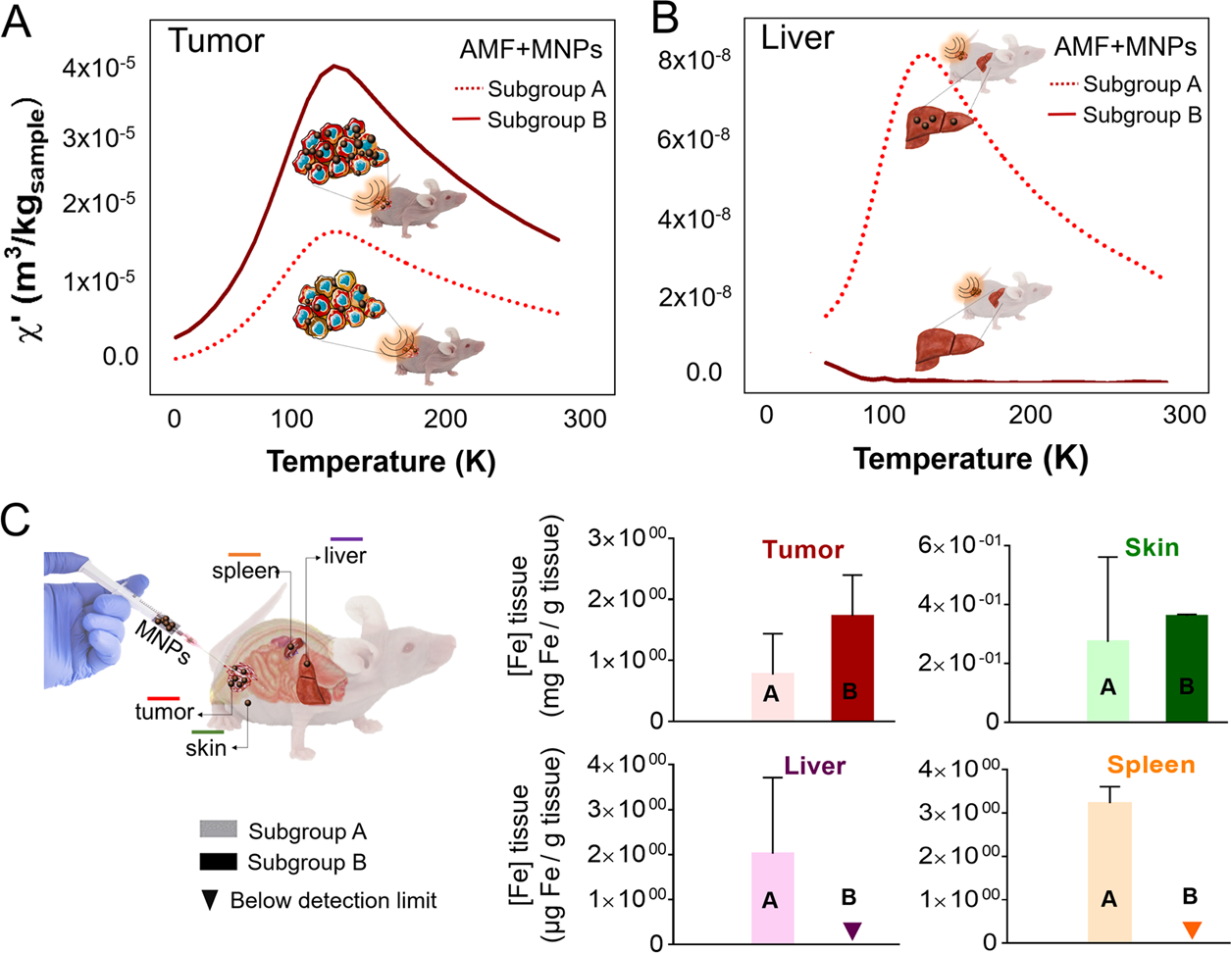
MNP biodistribution assessed by AC magnetic susceptibility 30 days after their intratumor injection. Temperature dependence of the in-phase susceptibility from the (A) tumor and (B) liver 30 days after the intratumor injection. (C) Average iron concentration (associated with MNPs) calculated from the magnetic characterization analysis for each of the analyzed tissues (tumor, liver, spleen, and skin next to the tumor) for the two subgroups of animals that received the complete treatment (AMF + MNPs). This figure was produced using images from the Servier Medical Art PPT image bank.

After sacrificing the mice at the end of the study, some organs were extracted (tumor, skin in contact with the tumor, liver, spleen, and blood) and the number of particles in each of them was analyzed using magnetic measurements. The temperature dependence of the AC magnetic susceptibility for all of the organs was measured and compared with that of the injected particles (Figure S1 from the Supporting Information). Previous works had validated this technique as a very sensitive way to detect the MNP biodistribution.^63−67^ The susceptibility maxima observed in Figure 6A,B agreed with the characterization of the particles and acted as a fingerprint of the presence of particles in a given tissue.^62,68^ The height of the out-of-phase susceptibility maxima (Figure S4 from the Supporting Information) was related to the number of particles within a given tissue and was used to quantify the iron concentration in the form of MNPs in each tissue sample.

The quantification process was performed in different tissues. However, as the tumor was split into several parts to be also analyzed by histology, some errors may have been introduced in the calculations of this particular tissue related to the heterogeneous MNP biodistribution, and results from the tumors have to be interpreted cautiously. In the tumors, an average concentration of iron in the form of MNPs of 0.7 ± 0.6 μg of iron/mg of tissue was found for subgroup A, while for subgroup B, this value was 2.5-fold higher (1.7 ± 0.6 μg of iron/mg of tissue), although it has to be considered that relatively big standard deviations were obtained in these tissues. Much lower iron concentrations were found in the rest of the analyzed tissues (Figure 6C).

To evaluate the percentage of iron from the initial administration that was found in each tissue, the whole tissue mass was considered to obtain the total iron content remaining in the tissues. In the tumors, this value was found to be quite heterogeneous (≈5–80 μg of iron, which corresponded to ≈2.5–40% of the mass of iron from the initial injection). In the skin samples, although still quite heterogeneous, a shorter range of iron amounts was found (≈3.5–17% of the mass of iron from the initial injection).

It was interesting to compare the different biodistribution patterns observed in animals that had a different response to MH treatment. Those animals that showed a weaker response (subgroup A) presented detectable amounts of particles in the liver and the spleen (total mass of iron: ≈3 μg of iron in the form of MNPs in the liver and ≈0.1 μg of iron in the spleen; iron concentrations per dry weight are shown in Figure 6C). In contrast, in animals belonging to subgroup B, with a better response to MH treatment, no particles in other organs (or at least under the detection limits of the technique) were found (Figures 6C and S4 from the Supporting Information). These data indicated that, in subgroup A, part of the injected dose had traveled from the tumor to other organs, reducing the potential effect of the treatment. The amount of particles found in the liver and spleen was significantly lower than in the tumor, as previously described by other authors.^56^ However, it should also be noted that it had been previously shown that the degradation of MNPs in these two organs, spleen and liver, can occur within this time frame,^63,66^ so the total number of nanoparticles that may have traveled to these organs was probably underestimated in our analysis.

One of the causes for the observation of MNPs in the liver and the spleen could be the extravasation of the particles through the tumor vasculature. Leakage of the MNPs toward blood vessels would result in their accumulation in the liver and spleen, the main organs for MNP accumulation after intravenous administration.^63,64,67^ In our case, no sign of MNPs was found in blood samples collected during the animal sacrifice, but this could be explained by a leakage at short times after the intravenous injection of MNPs.

Our results point toward the important effect that an inadequate biodistribution of MNPs in the body could have on the effectiveness of treatment. Therefore, we suggest that this kind of study be routinely performed after intratumor administration to verify that the intended number of particles remained in the tumor area, using whole-body *in vivo* imaging techniques such as magnetic resonance imaging (MRI) or magnetic particle imaging (MPI).

## Conclusions

Two different 3D cell culture models of the MIA PaCa-2 cell line, one with the particles just inside the cells and the other with the particles both inside and outside the cells, were prepared, and the MNP internalization and viability after the treatment were assessed for three different combinations of magnetic field amplitude and frequency. The In&Out model allowed us to evaluate the impact that the AMF exposure had on the MNP internalization. Furthermore, given that a different number of cells with internalized particles was achieved with each model, it was possible to relate the effectiveness of the treatment *in vitro* with the percentage of cells that contained MNPs. Finally, these 3D models allowed us to find the optimal AMF conditions, from those evaluated, to achieve the best treatment effectivity.

A xenograft murine model of pancreatic cancer was developed for the *in vivo* analysis of the optimized AMF conditions. CALR expression, a marker of the immune response, was significantly enhanced in those cells containing MNPs after the MH treatment, opening the way to further studies on immune stimulation after MH using animal models with a complete immune system. The histological analysis of tumor sections indicated a heterogeneous distribution of the particles within the tumor, with most of them being located in the outer areas. Nevertheless, the MH-treated mice showed the presence of significant amounts of particles in inner areas of the tumor. This is probably the result of the increased penetration of the particles toward the inner parts of the tumor after the exposure to several consecutive cycles of AMF. This observation points to future studies combining MH and chemotherapy to evaluate possible synergistic effects of both treatments, especially if the disruption of the extracellular matrix produced by the local heating effect of the noninternalized particles is also able to improve the permeability of drugs toward the inner parts of the tumor.

Finally, even though a decrease in the tumor growth rate was observed for all animals that received the complete MH treatment in comparison with the controls, it was possible to divide the animals treated into two subgroups depending on the differences found in their tumor growth rates. A clear relationship was then found between those subgroups and their whole-body particle biodistribution pattern. The subgroup of animals in which the treatment was less effective presented a lower number of particles in the tumor along with the presence of particles in the liver and the spleen. These results indicated the importance of correct biodistribution after the intratumor injection of the nanoparticles to achieve the most effective treatment.

In summary, our results revealed key parameters to be considered for the successful treatment of pancreatic cancer using magnetic hyperthermia. These results could be critical for the development of MH-based pancreatic cancer treatment schemes with higher therapeutic effectiveness.

## Materials and Methods

### Synthesis, Functionalization, and Characterization of Magnetic Nanoparticles

Iron oxide MNPs (≈11 nm) were synthesized by thermal decomposition in organic media based on a previously reported seed-mediated growth method^69^ using iron(III) acetylacetonate (Fe(acac)_3_) as a precursor.^63^ This procedure rendered oleic acid-coated hydrophobic MNPs, which were then transferred to water using a protocol based on the coating with an amphiphilic polymer (poly(maleic anhydride-alt-1-octadecene), PMAO, MW 30 000–50 000 Da) modified with TAMRA (tetramethylrhodamine 5(6)-carboxamide cadaverine (Anaspec, Seraing, Belgium, λ_ex_ = 543 nm, λ_em_ = 570 nm), a fluorophore that allows the *in vitro* tracking of the MNPs).^63^ Then, the coated MNPs were functionalized with glucose to provide further stability in biological media. The coated nanoparticles (1 mg of iron) were incubated with 42 μmol of *N*-(3-dimethylaminopropyl)-*N*′-ethylcarbodiimide hydrochloride (EDC) and 30 μmol of 4-aminophenyl β-d-glucopyranoside in 250 μL of sperm storage buffer (SSB) (50 mM boric acid and 50 mM sodium borate) at pH 9. After 3 h at room temperature, the excess of reagents was removed by washing the sample with phosphate-buffered saline (PBS) buffer at pH 7.4 in a centrifugal filter.^70^ Finally, nanoparticles were passed through syringe filters with a pore size of 0.22 μm (Merck Millipore, Darmstadt, Germany). Dynamic light-scattering and ζ-potential measurements were performed in water and in complete Dulbecco’s modified Eagle’s medium GlutaMAX Supplement (cDMEM; Gibco, Thermo Fisher Scientific) on a Malvern Zetasizer Nano-ZS, using 10 runs per measurement and five replicates at 25 °C and pH 7. Particle size and morphology were studied by transmission electron microscopy (TEM) using a Tecnai T20 (FEI company, OR) microscope operating at 200 kV. The sample was prepared by placing a drop of a diluted suspension of the MNPs in water onto a carbon-coated grid and allowing it to dry. Particle size was determined by manual measurement of 200 particles using Digital Micrograph software. The heating capacity of the MNPs was determined using a commercial alternating magnetic field generator (DM100; Nanoscale Biomagnetics, Spain). A 1 mg Fe/mL MNP suspension was placed in a closed container centered in the inductive coil. The AMF was applied for 5 min using a field amplitude of *H* = 20 kA/m and a frequency of 829 kHz, while the temperature was recorded using an optic fiber sensor incorporated in the equipment. For the magnetic characterization, the MNP liquid sample was allowed to dry at room temperature deposited in a piece of cotton wool that was subsequently placed in a gelatin capsule. An additional sample of the particles dispersed in agar and then freeze-dried was prepared to be used in the quantification protocol. AC magnetic susceptibility measurements were performed in a Quantum Design MPMS-XL SQUID magnetometer with an alternating current (AC) option. Measurements were acquired using a field amplitude of 0.41 Oe and a frequency of 11 Hz in the temperature range between 5 and 300 K.

### Cell Culture

The MIA PaCa-2 (ATCC CRL-1420) pancreatic cancer cell line was cultured and maintained in complete Dulbecco’s modified Eagle’s medium GlutaMAX Supplement (cDMEM; Gibco, Thermo Fisher Scientific) supplemented with 10% fetal bovine serum (FBS, Invitrogen), 100 U/mL penicillin G (sodium salt), and 100 μg/mL streptomycin sulfate (Invitrogen) at 37 °C in a humidified atmosphere at 5% CO_2_. Every 3 or 4 days depending on the confluency, the cell culture was diluted to 1:10. To detach the cells, they were incubated with Trypsin ethylenediaminetetraacetic acid (EDTA) solution (Sigma-Aldrich) for 4 min at 37 °C. Finally, cells were collected in fresh cDMEM. To prepare the 3D cell culture models, two different strategies of incubation with MNPs were followed based on a previously reported method.^6^ The first model where nanoparticles are located only inside the cells—In Model—was prepared by incubation of the cells, cultured in monolayer conditions, with MNPs (0.2 mg Fe/mL) for 24 h at 37 °C. After this time, the medium with MNPs was discarded and the cells were detached and washed by centrifugation (300*g*, 5 min) twice and then were used to form the collagen structure (rat tail collagen type I (protein concentration 2.05 mg/mL in 0.6% acetic acid, First Link (UK) Ltd.)). Just after the collagen gelling, 0.5 mL of cDMEM was added to the 3D structure. The collagen gelling process takes 20 min, and after that, the 3D culture is maintained at 37 °C for 2.5 h before the MH experiments to stabilize the cells in the 3D structure. In the other model where nanoparticles are located both inside and outside the cells—In&Out Model—the 3D cell culture was generated first with the cells in suspension, and then, 0.5 mL of an MNP suspension (0.2 mg Fe/mL) in cDMEM was added and incubated at 37 °C for 24 h. After the incubation time, the supernatant was removed, the 3D cell culture was washed twice, and 0.5 mL of complete culture medium was added to the 3D model. The MH application starts just after this process.

### Tumor Xenograft Model

Pathogen-free male athymic nude mice (Crl:NU(NCr-*Foxn1*^*nu*^)), 6 weeks old, received a single subcutaneous injection into the right flank with the human pancreatic cancer cell line MIA PaCa-2-2 (5 × 10^6^ cells) suspended in 0.2 mL of sterile DMEM culture medium without phenol red using a 25 G needle. During the cell injection, animals were anesthetized by inhalation of isoflurane (4% for the induction step and 2% for maintenance). The mice were commercially obtained from Charles River Laboratory and were maintained in the animal facilities of the Centro de Investigaciones Biomédicas de Arago′n, CIBA (Instituto Aragonés de Ciencias de la Salud (IACS), Universidad de Zaragoza). Before any procedure, mice were held for one week after arriving from the animal facilities for acclimation. Animals were maintained according to the institutional animal use and care regulation of CIBA. All animal experiments were conducted according to the RD53/2013 law and approved by the Ethics Committee for animal experiments from the University of Zaragoza, which is an accredited animal welfare body.

### Magnetic Hyperthermia Treatment

A commercial AMF generator (DM3, nB nanoscale Biomagnetics, Zaragoza, Spain) housed inside the animal facilities was used in all scenarios. The exposure time to AMF was 30 min in each case. *In vitro*, MIA PaCa-2 cells cultured in 3D conditions in 24-well plates were thermalized at 37 °C using a water bath pump (Stryker, Medical Devices & Equipment Manufacturing Company) connected to a water tubing jacket. Then, cells were exposed to the AMF using different schemes of frequency and field amplitude: AMF 1 (377 kHz, 13 kA/m), AMF 2 (110 kHz, 31.9 kA/m), and AMF 3 (228 kHz, 23.9 kA/m). *In vivo*, immunodeficient mice were inoculated with human pancreatic cancer cells as previously described. About 3 weeks later, when the tumor size was about 0.1 cm^3^, 3 mg/mL MNPs in a final volume of 0.05 mL per tumor were injected intratumorally using a 30 G needle. Mice were divided randomly into four different groups (control, tumor control; AMF, tumor exposed to AMF; MNP, tumor injected with MNPs; AMF + MNPs, tumor injected with MNPs and exposed to the AMF). On the same day of MNP injection and on the following 2 days, mice were exposed to the AMF (*f* = 196 kHz; *H* = 26 kA/m), the closest AMF conditions to AMF 3 tested in the *in vitro* experiments available in our device that resulted in an *f* × *H*_0_ value of 5 × 10^–9^ A m^–1^ s^–1^. Mice were anesthetized with isoflurane and maintained during the AMF exposure on a hot water bath system that prevents the mice from suffering hypothermia. The rectal temperature was registered during the AMF exposure to control the general state of the animals. After the last AMF exposure, mice were maintained to evaluate the response to the treatment. Tumor dimensions (length, width, and height) were measured twice a week with a digital caliper. The weight of mice was followed during the experiment as an indicator of their wellness.

### Confocal Microscopy

To study the MNP cell internalization, the 3D cell cultures were fixed for 20 min with 0.5 mL of paraformaldehyde (4%). Then, the nuclei were stained with 4′,6-diamidino-2-phenylindole (DAPI) and the cytoskeleton with Phalloidin 488. A Zeiss LSM 880 confocal microscope equipped with a 63x/1.40 Plan Apochromat objective was used to acquire the images of the cells. The laser sources used were 458 nm, 488 nm (argon ion), and 561 nm (DPSS, diode-pumped solid state). The ZEN microscope as well as imaging software were used for the image analysis.

### Flow Cytometry Studies

All samples were analyzed in a Gallios flow cytometer (Beckman Coulter), and the data were interpreted with Kaluza 2.1 software (Beckman Coulter).

### *In Vitro* Assays

Cells were released from the 3D cell cultures using a treatment with collagenase type I (isolated from *Clostridium histolyticum* lyophilized, nonsterile, Gibco Thermo Fisher Scientific) at 2 mg/mL in Hank’s balanced salt solution (HBSS) for 30 min at 37 °C. Then, in each case, cells were washed by centrifugation (300*g*, 5 min) and resuspended in PBS (pH = 7.4). To determine the MNP uptake, cells resuspended in PBS at a concentration of 2.5 × 10^4^ cells/mL were analyzed by flow cytometry in the FL2 channel at 575 nm. To study the cell viability, cells were resuspended in 1× Annexin V binding buffer (10 mM 4-(2-hydroxyethyl)-1-piperazineethanesulfonic acid (HEPES)/NaOH (pH = 7.4) 140 mM NaCl, 2.5 mM CaCl_2_) at a concentration of 10^6^ cells/mL. Then, 5 × 10^–3^ mL of Annexin V-FITC (fluorescein isothiocyanate, λ_ex_ = 488 nm, λ_em_ = 530 nm) and 5 × 10^–3^ mL of propidium iodide (PI, λ_ex_ = 535 nm, λ_em_ = 617 nm) were added to 0.1 mL of the cell suspension and incubated at room temperature for 15 min in the dark (FITC-Annexin V apoptosis detection kit). After the incubation period, 0.4 mL of 1× Annexin binding buffer was added and the sample was analyzed by flow cytometry.

### *Ex Vivo* Assays

Subcutaneous tumors from euthanized mice were placed into a corning tube with the cDMEM medium. Then, on a glass surface, each tumor was disengaged in small pieces with scalpels and transferred into a 15 mL corning tube with 3 mL of collagenase P (1.5 mg/mL). The mixture was placed in continuous rotation for 10–12 min in an incubator at 37 °C. The reaction was stopped by addition of 1 mL of FBS and cDMEM until a final volume of 10 mL, and then, the sample was centrifuged for 5 min at 300*g*. The supernatant was removed, and 2 mL of trypsin/EDTA was added to the pellet and mixed gently for 3 min at 37 °C. The trypsin was neutralized by addition of 2 mL of FBS and cDMEM until a final volume of 15 mL. After a thorough mixing, the suspension was centrifuged for 5 min at 300*g* and the supernatant was discarded. The pellet was resuspended in 5 mL of cDMEM and filtered through a 100 μm filter and then through a 40 μm strainer into a 50 mL corning tube. The sample was transferred into a 15 mL tube and centrifuged for 5 min at 300*g*. The supernatant was discarded, and the pellet was resuspended in 0.8 mL of 1× red blood cell (RBC) lysis buffer (Invitrogen by Thermo Fisher Scientific) by continuously agitating with the pipette for 20–30 s. The reaction was stopped by addition of a DMEM medium (5–10 mL), and then, the mixture was washed by centrifugation (5 min at 300*g*). The supernatant was discarded and resuspended in PBS. To evaluate immunogenic death signals, cells from tumor samples, from two animals in each group, were resuspended in 1× binding buffer at a concentration of 1 × 10^4^ cells/mL. Then, 2 × 10^–3^ mL of α-Calreticulin antibody-Alexa Fluor 405 (Abcam ab210431) was added to 0.05 mL of the cell suspension and incubated at room temperature for 30 min in the dark. After the incubation period, the cells were washed three times by centrifugation (300*g*, 5 min). Finally, 0.4 mL of 1× binding buffer was added and the sample was analyzed by flow cytometry. To determine the MNP uptake, the cells were analyzed in the FL2 channel at 575 nm.

### Mice Sample Preparation and Analysis

Mice were euthanized by CO_2_ inhalation, and blood was directly extracted from the heart. In addition, the tumors, the skin next to them, the livers, and the spleens were removed. Tumor pieces were fixed in 4% paraformaldehyde (PFA) and processed to perform hematoxylin/eosin and Perls Prussian blue staining. Cell death was assessed by analysis of morphological changes. All of the sample preparation including the scanning in the light field were performed by the “Servicio Científico Te′cnico-Microscopía y Anatomi′a Patolo′gica” of the CIBA (IACS, Universidad de Zaragoza). For the magnetic measurements, tissue samples (liver, spleen, skin, and tumor pieces) were freeze-dried in Telstar cryodos-50 for 24 h and placed directly into gelatin capsules for their characterization. Magnetic susceptibility measurements were performed in a Quantum Design MPMS-XL SQUID magnetometer with an alternating current (AC) amplitude of 0.41 Oe, in the temperature range between 5 and 300 K and at a frequency of 11 Hz.

### Statistical Analysis

All data were expressed as mean ± SD of a minimum of three biological replicas. Statistical significance of difference in means was evaluated using GraphPad Prism v7.00. Two-way ANOVA and one-way ANOVA tests were used for the analysis of the data. The confidence interval was 95%. Sidak’s and Dunnet′s multiple comparison post tests were used to determine the means that differed.

## Supporting Information Available

Characterization of the magnetic nanoparticles (Figure S1); cell viability studies (Figure S2); histological assessment of tumor sections (Figure S3); magnetic characterization of tissue samples (Figure S4) (PDF)

## References

[ref1] Kanat O.; Ertas H. Shattering the Castle Walls: Anti-Stromal Therapy for Pancreatic Cancer. World J. Gastrointest. Oncol. 2018, 10, 202–210. 10.4251/wjgo.v10.i8.202. 30147846 PMC6107476

[ref2] Elahi-Gedwillo K. Y.; Carlson M.; Zettervall J.; Provenzano P. P. Antifibrotic Therapy Disrupts Stromal Barriers and Modulates the Immune Landscape in Pancreatic Ductal Adenocarcinoma. Cancer Res. 2019, 79, 372–386. 10.1158/0008-5472.CAN-18-1334. 30401713 PMC6335156

[ref3] van Mackelenbergh M. G.; Stroes C. I.; Spijker R.; van Eijck C. H.; Wilmink J. W.; Bijlsma M. F.; van Laarhoven H. W. Clinical Trials Targeting the Stroma in Pancreatic Cancer: A Systematic Review and Meta-Analysis. Cancers 2019, 11, 588 10.3390/cancers11050588. PMC6562438 31035512

[ref4] Bogart L. K.; Pourroy G.; Murphy C. J.; Puntes V.; Pellegrino T.; Rosenblum D.; Peer D.; Lévy R. Nanoparticles for Imaging, Sensing, and Therapeutic Intervention. ACS Nano 2014, 8, 3107–3122. 10.1021/nn500962q. 24641589 PMC4123720

[ref5] Brachi G.; Bussolino F.; Ciardelli G.; Mattu C. Nanomedicine for Imaging and Therapy of Pancreatic Adenocarcinoma. Front. Bioeng. Biotechnol. 2019, 7, 307 10.3389/fbioe.2019.00307. 31824928 PMC6880757

[ref6] Beola L.; Asín L.; Fratila R. M.; Herrero V.; de la Fuente J. M.; Grazú V.; Gutiérrez L. Dual Role of Magnetic Nanoparticles as Intracellular Hotspots and Extracellular Matrix Disruptors Triggered by Magnetic Hyperthermia in 3d Cell Culture Models. ACS Appl. Mater. Interfaces 2018, 10, 44301–44313. 10.1021/acsami.8b18270. 30480993

[ref7] Hervault A.; Thanh N. T. K. Magnetic Nanoparticle-Based Therapeutic Agents for Thermo-Chemotherapy Treatment of Cancer. Nanoscale 2014, 6, 11553–11573. 10.1039/C4NR03482A. 25212238

[ref8] Maier-Hauff K.; Ulrich F.; Nestler D.; Niehoff H.; Wust P.; Thiesen B.; Orawa H.; Budach V.; Jordan A. Efficacy and Safety of Intratumoral Thermotherapy Using Magnetic Iron-Oxide Nanoparticles Combined with External Beam Radiotherapy on Patients with Recurrent Glioblastoma Multiforme. J. Neuro-Oncol. 2011, 103, 317–324. 10.1007/s11060-010-0389-0. PMC3097345 20845061

[ref9] Johannsen M.; Thiesen B.; Wust P.; Jordan A. Magnetic Nanoparticle Hyperthermia for Prostate Cancer. Int. J. Hyperthermia 2010, 26, 790–795. 10.3109/02656731003745740. 20653418

[ref10] Kettering M.; Grau I.; Pömpner N.; Stapf M.; Gajda M.; Teichgräber U.; Hilger I. Means to Increase the Therapeutic Efficiency of Magnetic Heating of Tumors. Biomed. Tech. 2015, 60, 505–517. 10.1515/bmt-2015-0052. 26351784

[ref11] Kossatz S.; Ludwig R.; Dähring H.; Ettelt V.; Rimkus G.; Marciello M.; Salas G.; Patel V.; Teran F. J.; Hilger I. High Therapeutic Efficiency of Magnetic Hyperthermia in Xenograft Models Achieved with Moderate Temperature Dosages in the Tumor Area. Pharm. Res. 2014, 31, 3274–3288. 10.1007/s11095-014-1417-0. 24890197 PMC4224751

[ref12] Piehler S.; Wucherpfennig L.; Tansi F. L.; Berndt A.; Quaas R.; Teichgraeber U. K.; Hilger I. Hyperthermia Affects Collagen Fiber Architecture and Induces Apoptosis in Pancreatic and Fibroblast Tumor Hetero-Spheroids in Vitro. Nanomedicine 2020, 28, 102183 10.1016/j.nano.2020.102183. 32222478

[ref13] Wang L.; Dong J.; Ouyang W.; Wang X.; Tang J. Anticancer Effect and Feasibility Study of Hyperthermia Treatment of Pancreatic Cancer Using Magnetic Nanoparticles. Oncol. Rep. 2012, 27, 719–726. 10.3892/or.2011.1567. 22134718

[ref14] Ludwig R.; Teran F. J.; Teichgraeber U.; Hilger I. Nanoparticle-Based Hyperthermia Distinctly Impacts Production of Ros, Expression of Ki-67, Top2a, and Tpx2, and Induction of Apoptosis in Pancreatic Cancer. Int. J. Nanomed. 2017, 12, 1009–1018. 10.2147/IJN.S108577. PMC5304998 28223795

[ref15] Attaluri A.; Kandala S. K.; Zhou H.; Wabler M.; DeWeese T. L.; Ivkov R. Magnetic Nanoparticle Hyperthermia for Treating Locally Advanced Unresectable and Borderline Resectable Pancreatic Cancers: The Role of Tumor Size and Eddy-Current Heating. Int. J. Hyperthermia 2020, 37, 108–119. 10.1080/02656736.2020.1798514. 33426990 PMC8363047

[ref16] Mejías R.; Hernández Flores P.; Talelli M.; Tajada-Herráiz J. L.; Brollo M. E.; Portilla Y.; Morales M. P.; Barber D. F. Cell-Promoted Nanoparticle Aggregation Decreases Nanoparticle-Induced Hyperthermia under an Alternating Magnetic Field Independently of Nanoparticle Coating, Core Size, and Subcellular Localization. ACS Appl. Mater. Interfaces 2018, 11, 340–355. 10.1021/acsami.8b18451. 30525392

[ref17] Balakrishnan P. B.; Silvestri N.; Fernandez-Cabada T.; Marinaro F.; Fernandes S.; Fiorito S.; Miscuglio M.; Serantes D.; Ruta S.; Livesey K.; et al. Exploiting Unique Alignment of Cobalt Ferrite Nanoparticles, Mild Hyperthermia, and Controlled Intrinsic Cobalt Toxicity for Cancer Therapy. Adv. Mater. 2020, 32, 2003712 10.1002/adma.202003712. 33002227

[ref18] Conde-Leboran I.; Baldomir D.; Martinez-Boubeta C.; Chubykalo-Fesenko O.; del Puerto Morales M.; Salas G.; Cabrera D.; Camarero J.; Teran F. J.; Serantes D. A Single Picture Explains Diversity of Hyperthermia Response of Magnetic Nanoparticles. J. Phys. Chem. C 2015, 119, 15698–15706. 10.1021/acs.jpcc.5b02555.

[ref19] Krysko D. V.; Garg A. D.; Kaczmarek A.; Krysko O.; Agostinis P.; Vandenabeele P. Immunogenic Cell Death and Damps in Cancer Therapy. Nat. Rev. Cancer 2012, 12, 860–875. 10.1038/nrc3380. 23151605

[ref20] Obeid M.; Tesniere A.; Ghiringhelli F.; Fimia G. M.; Apetoh L.; Perfettini J.-L.; Castedo M.; Mignot G.; Panaretakis T.; Casares N.; et al. Calreticulin Exposure Dictates the Immunogenicity of Cancer Cell Death. Nat. Med. 2007, 13, 54–61. 10.1038/nm1523. 17187072

[ref21] Liu X.; Zheng J.; Sun W.; Zhao X.; Li Y.; Gong N.; Wang Y.; Ma X.; Zhang T.; Zhao L.-Y.; et al. Ferrimagnetic Vortex Nanoring-Mediated Mild Magnetic Hyperthermia Imparts Potent Immunological Effect for Treating Cancer Metastasis. ACS Nano 2019, 13, 8811–8825. 10.1021/acsnano.9b01979. 31328922

[ref22] Houg D. S.; Bijlsma M. F. The Hepatic Pre-Metastatic Niche in Pancreatic Ductal Adenocarcinoma. Mol. Cancer 2018, 17, 1–18. 10.1186/s12943-018-0842-9. 29903049 PMC6003100

[ref23] Chao Y.; Chen G.; Liang C.; Xu J.; Dong Z.; Han X.; Wang C.; Liu Z. Iron Nanoparticles for Low-Power Local Magnetic Hyperthermia in Combination with Immune Checkpoint Blockade for Systemic Antitumor Therapy. Nano Lett. 2019, 19, 4287–4296. 10.1021/acs.nanolett.9b00579. 31132270

[ref24] Wilhelm S.; Tavares A. J.; Dai Q.; Ohta S.; Audet J.; Dvorak H. F.; Chan W. C. Analysis of Nanoparticle Delivery to Tumours. Nat. Rev. Mater. 2016, 1, 16014 10.1038/natrevmats.2016.14.

[ref25] Beola L.; Gutiérrez L.; Grazú V.; Asín L. A Roadmap to the Standardization of in Vivo Magnetic Hyperthermia. In Nanomaterials for Magnetic and Optical Hyperthermia Applications; Elsevier, 2019; pp 317–337.

[ref26] Beola L. L.; Asín L.; Roma-Rodrigues C.; Fernández-Afonso Y.; Fratila R. M. M.; Serantes D.; Ruta S.; Chantrell R.; Fernandes A. R.; Baptista P. V.; et al. Intracellular Number of Magnetic Nanoparticles Modulates the Apoptotic Death Pathway after Magnetic Hyperthermia Treatment. ACS Appl. Mater. Interfaces 2020, 12, 43474–43487. 10.1021/acsami.0c12900. 32870658

[ref27] Moros M.; Hernáez B.; Garet E.; Dias J. T.; Sáez B.; Grazú V.; González-Fernández A.; Alonso C.; de la Fuente J. M. Monosaccharides Versus Peg-Functionalized Nps: Influence in the Cellular Uptake. ACS Nano 2012, 6, 1565–1577. 10.1021/nn204543c. 22214244

[ref28] Hergt R.; Dutz S. Magnetic Particle Hyperthermia—Biophysical Limitations of a Visionary Tumour Therapy. J. Magn. Magn. Mater. 2007, 311, 187–192. 10.1016/j.jmmm.2006.10.1156.

[ref29] Dutz S.; Hergt R. Magnetic Nanoparticle Heating and Heat Transfer on a Microscale: Basic Principles, Realities and Physical Limitations of Hyperthermia for Tumour Therapy. Int. J. Hyperthermia 2013, 29, 790–800. 10.3109/02656736.2013.822993. 23968194

[ref30] Dias J. T.; Moros M.; Del Pino P.; Rivera S.; Grazu V.; de la Fuente J. M. DNA as a Molecular Local Thermal Probe for the Analysis of Magnetic Hyperthermia. Angew. Chem., Int. Ed. 2013, 52, 11526–11529. 10.1002/anie.201305835. 24115553

[ref31] Rodrigues M.; Piñol R.; Antorrena G.; Brites C. D.; Silva N. J.; Murillo J. L.; Cases R.; Díez I.; Palacio F.; Torras N.; et al. Luminescent Thermometers: Implementing Thermometry on Silicon Surfaces Functionalized by Lanthanide-Doped Self-Assembled Polymer Monolayers. Adv. Funct. Mater. 2016, 26, 312 10.1002/adfm.201670015.

[ref32] Serantes D.; Baldomir D.; Martinez-Boubeta C.; Simeonidis K.; Angelakeris M.; Natividad E.; Castro M.; Mediano A.; Chen D.-X.; Sanchez A.; et al. Influence of Dipolar Interactions on Hyperthermia Properties of Ferromagnetic Particles. J. Appl. Phys. 2010, 108, 073918 10.1063/1.3488881.

[ref33] Hayashi K.; Nakamura M.; Miki H.; Ozaki S.; Abe M.; Matsumoto T.; Sakamoto W.; Yogo T.; Ishimura K. Magnetically Responsive Smart Nanoparticles for Cancer Treatment with a Combination of Magnetic Hyperthermia and Remote-Control Drug Release. Theranostics 2014, 4, 834–844. 10.7150/thno.9199. 24955144 PMC4063981

[ref34] Hoopes P. J.; Mazur C. M.; Osterberg B.; Song A.; Gladstone D. J.; Steinmetz N. F.; Veliz F. A.; Bursey A. A.; Wagner R. J.; Fiering S. N. In Effect of Intra-Tumoral Magnetic NanoparticleHyperthermia and Viral Nanoparticle Immunogenicity on Primary and Metastatic Cancer, Energy-based Treatment of Tissue and Assessment IX. International Society for Optics and Photonics, 2017; pp 100660G-1–100660G-8. 10.1117/12.2256062 PMC5711520 29203952

[ref35] Zhang Z.-Q.; Song S.-C. Thermosensitive/Superparamagnetic Iron Oxide Nanoparticle-Loaded Nanocapsule Hydrogels for Multiple Cancer Hyperthermia. Biomaterials 2016, 106, 13–23. 10.1016/j.biomaterials.2016.08.015. 27543919

[ref36] Guisasola E.; Asín L.; Beola L.; de la Fuente J. M.; Baeza A.; Vallet-Regí M. Beyond Traditional Hyperthermia: In Vivo Cancer Treatment with Magnetic-Responsive Mesoporous Silica Nanocarriers. ACS Appl. Mater. Interfaces 2018, 10, 12518–12525. 10.1021/acsami.8b02398. 29561590

[ref37] Kolosnjaj-Tabi J.; Marangon I.; Nicolas-Boluda A.; Silva A. K.; Gazeau F. Nanoparticle-Based Hyperthermia, a Local Treatment Modulating the Tumor Extracellular Matrix. Pharmacol. Res. 2017, 126, 123–137. 10.1016/j.phrs.2017.07.010. 28720518

[ref38] Kroemer G.; Galluzzi L.; Kepp O.; Zitvogel L. Immunogenic Cell Death in Cancer Therapy. Annu. Rev. Immunol. 2013, 31, 51–72. 10.1146/annurev-immunol-032712-100008. 23157435

[ref39] Garg A. D.; Dudek-Peric A. M.; Romano E.; Agostinis P. Immunogenic Cell Death. Int. J. Dev. Biol. 2015, 59, 131–140. 10.1387/ijdb.150061pa. 26374534

[ref40] Gradiz R.; Silva H. C.; Carvalho L.; Botelho M. F.; Mota-Pinto A. Mia Paca-2 and Panc-1–Pancreas Ductal Adenocarcinoma Cell Lines with Neuroendocrine Differentiation and Somatostatin Receptors. Sci. Rep. 2016, 6, 21648 10.1038/srep21648. 26884312 PMC4756684

[ref41] Linton S. S.; Abraham T.; Liao J.; Clawson G. A.; Butler P. J.; Fox T.; Kester M.; Matters G. L. Tumor-Promoting Effects of Pancreatic Cancer Cell Exosomes on Thp-1-Derived Macrophages. PLoS One 2018, 13, e0206759 10.1371/journal.pone.0206759. 30383833 PMC6211741

[ref42] Chen Y.-H.; Chen Y.-C.; Lin C.-C.; Hsieh Y.-P.; Hsu C.-S.; Hsieh M.-C. Synergistic Anticancer Effects of Gemcitabine with Pitavastatin on Pancreatic Cancer Cell Line Mia Paca-2 in Vitro and in Vivo. Cancer Manage. Res. 2020, 12, 4645–4665. 10.2147/CMAR.S247876. PMC7306478 32606957

[ref43] Dixon S. J.; Lemberg K. M.; Lamprecht M. R.; Skouta R.; Zaitsev E. M.; Gleason C. E.; Patel D. N.; Bauer A. J.; Cantley A. M.; Yang W. S.; et al. Ferroptosis: An Iron-Dependent Form of Nonapoptotic Cell Death. Cell 2012, 149, 1060–1072. 10.1016/j.cell.2012.03.042. 22632970 PMC3367386

[ref44] Shen Z.; Liu T.; Li Y.; Lau J.; Yang Z.; Fan W.; Zhou Z.; Shi C.; Ke C.; Bregadze V. I.; et al. Fenton-Reaction-Acceleratable Magnetic Nanoparticles for Ferroptosis Therapy of Orthotopic Brain Tumors. ACS Nano 2018, 12, 11355–11365. 10.1021/acsnano.8b06201. 30375848

[ref45] Wang S.; Luo J.; Zhang Z.; Dong D.; Shen Y.; Fang Y.; Hu L.; Liu M.; Dai C.; Peng S. Iron and Magnetic: New Research Direction of the Ferroptosis-Based Cancer Therapy. Am. J. Cancer Res. 2018, 8, 1933–1946. 30416846 PMC6220147

[ref46] Yu B.; Choi B.; Li W.; Kim D.-H. Magnetic Field Boosted Ferroptosis-Like Cell Death and Responsive Mri Using Hybrid Vesicles for Cancer Immunotherapy. Nat. Commun. 2020, 11, 3637 10.1038/s41467-020-17380-5. 32686685 PMC7371635

[ref47] Guo Y.; Ran Y.; Wang Z.; Cheng J.; Cao Y.; Yang C.; Liu F.; Ran H. Magnetic-Responsive and Targeted Cancer Nanotheranostics by Pa/Mr Bimodal Imaging-Guided Photothermally Triggered Immunotherapy. Biomaterials 2019, 219, 119370 10.1016/j.biomaterials.2019.119370. 31357006

[ref48] Liu X.; Yan B.; Li Y.; Ma X.; Jiao W.; Shi K.; Zhang T.; Chen S.; He Y.; Liang X.-J.; Fan H. Graphene Oxide-Grafted Magnetic Nanorings Mediated Magnetothermodynamic Therapy Favoring Reactive Oxygen Species-Related Immune Response for Enhanced Antitumor Efficacy. ACS Nano 2020, 14, 1936–1950. 10.1021/acsnano.9b08320. 31961656

[ref49] Zhang N.; Song J.; Liu Y.; Liu M.; Zhang L.; Sheng D.; Deng L.; Yi H.; Wu M.; Zheng Y.; et al. Photothermal Therapy Mediated by Phase-Transformation Nanoparticles Facilitates Delivery of Anti-Pd1 Antibody and Synergizes with Antitumor Immunotherapy for Melanoma. J. Controlled Release 2019, 306, 15–28. 10.1016/j.jconrel.2019.05.036. 31132380

[ref50] Korangath P.; Barnett J. D.; Sharma A.; Henderson E. T.; Stewart J.; Yu S.-H.; Kandala S. K.; Yang C.-T.; Caserto J. S.; Hedayati M.; et al. Nanoparticle Interactions with Immune Cells Dominate Tumor Retention and Induce T Cell–Mediated Tumor Suppression in Models of Breast Cancer. Sci. Adv. 2020, 6, eaay1601 10.1126/sciadv.aay1601. 32232146 PMC7096167

[ref51] Netea M. G.; Joosten L. A.; Latz E.; Mills K. H.; Natoli G.; Stunnenberg H. G.; O’Neill L. A.; Xavier R. J. Trained Immunity: A Program of Innate Immune Memory in Health and Disease. Science 2016, 352, aaf1098-1–aaf1098-9. 10.1126/science.aaf1098. 27102489 PMC5087274

[ref52] Kossatz S.; Grandke J.; Couleaud P.; Latorre A.; Aires A.; Crosbie-Staunton K.; Ludwig R.; Dähring H.; Ettelt V.; Lazaro-Carrillo A.; Calero M.; Sader M.; Courty J.; Volkov Y.; Prina-Mello A.; Villanueva A.; Somoza Á.; Cortajarena A. L.; Miranda R.; Hilger I. Efficient Treatment of Breast Cancer Xenografts with Multifunctionalized Iron Oxide Nanoparticles Combining Magnetic Hyperthermia and Anti-Cancer Drug Delivery. Breast Cancer Res. 2015, 17, 66 10.1186/s13058-015-0576-1. 25968050 PMC4451751

[ref53] Brusentsov N. A.; Nikitin L. V.; Brusentsova T. N.; Kuznetsov A. A.; Bayburtskiy F. S.; Shumakov L. I.; Jurchenko N. Y. Magnetic Fluid Hyperthermia of the Mouse Experimental Tumor. J. Magn. Magn. Mater. 2002, 252, 378–380. 10.1016/S0304-8853(02)00634-0.

[ref54] Espinosa A.; Di Corato R.; Kolosnjaj-Tabi J.; Flaud P.; Pellegrino T.; Wilhelm C. Duality of Iron Oxide Nanoparticles in Cancer Therapy: Amplification of Heating Efficiency by Magnetic Hyperthermia and Photothermal Bimodal Treatment. ACS Nano 2016, 10, 2436–2446. 10.1021/acsnano.5b07249. 26766814

[ref55] Chen Y.; Jiang L.; Wang R.; Lu M.; Zhang Q.; Zhou Y.; Wang Z.; Lu G.; Liang P.; Ran H.; Chen H.; Zheng Y. Injectable Smart Phase-Transformation Implants for Highly Efficient in Vivo Magnetic-Hyperthermia Regression of Tumors. Adv. Mater. 2014, 26, 7468–7473. 10.1002/adma.201402509. 25167961

[ref56] Kettering M.; Richter H.; Wiekhorst F.; Bremer-Streck S.; Trahms L.; Kaiser W. A.; Hilger I. Minimal-Invasive Magnetic Heating of Tumors Does Not Alter Intra-Tumoral Nanoparticle Accumulation, Allowing for Repeated Therapy Sessions: An in Vivo Study in Mice. Nanotechnology 2011, 22, 505102 10.1088/0957-4484/22/50/505102. 22107782

[ref57] Giustini A.; Ivkov R.; Hoopes P. Magnetic Nanoparticle Biodistribution Following Intratumoral Administration. Nanotechnology 2011, 22, 345101 10.1088/0957-4484/22/34/345101. 21795772 PMC3158492

[ref58] Javidi M.; Heydari M.; Karimi A.; Haghpanahi M.; Navidbakhsh M.; Razmkon A. Evaluation of the Effects of Injection Velocity and Different Gel Concentrations on Nanoparticles in Hyperthermia Therapy. J. Biomed. Phys. Eng. 2014, 4, 151–162. 25599061 PMC4289522

[ref59] Kuboyabu T.; Yabata I.; Aoki M.; Banura N.; Nishimoto K.; Mimura A.; Murase K. Magnetic Particle Imaging for Magnetic Hyperthermia Treatment: Visualization and Quantification of the Intratumoral Distribution and Temporal Change of Magnetic Nanoparticles in Vivo. Open J. Med. Imaging 2016, 6, 1–15. 10.4236/ojmi.2016.61001.

[ref60] Attaluri A.; Ma R.; Qiu Y.; Li W.; Zhu L. Nanoparticle Distribution and Temperature Elevations in Prostatic Tumours in Mice During Magnetic Nanoparticle Hyperthermia. Int. J. Hyperthermia 2011, 27, 491–502. 10.3109/02656736.2011.584856. 21756046

[ref61] Stylianopoulos T.; Martin J. D.; Chauhan V. P.; Jain S. R.; Diop-Frimpong B.; Bardeesy N.; Smith B. L.; Ferrone C. R.; Hornicek F. J.; Boucher Y.; et al. Causes, Consequences, and Remedies for Growth-Induced Solid Stress in Murine and Human Tumors. Proc. Natl. Acad. Sci. U.S.A. 2012, 109, 15101–15108. 10.1073/pnas.1213353109. 22932871 PMC3458380

[ref62] López A.; Gutierrez L.; Lazaro F. J. The Role of Dipolar Interaction in the Quantitative Determination of Particulate Magnetic Carriers in Biological Tissues. Phys. Med. Biol. 2007, 52, 5043–5056. 10.1088/0031-9155/52/16/022. 17671352

[ref63] Stepien G.; Moros M.; Perez-Hernandez M.; Monge M.; Gutierrez L.; Fratila R. M.; Las Heras M.; Menao Guillen S.; Puente Lanzarote J. J.; Solans C.; Pardo J.; de la Fuente J. M. Effect of Surface Chemistry and Associated Protein Corona on the Long-Term Biodegradation of Iron Oxide Nanoparticles in Vivo. ACS Appl. Mater. Interfaces 2018, 10, 4548–4560. 10.1021/acsami.7b18648. 29328627

[ref64] Rojas J. M.; Gavilan H.; Del Dedo V.; Lorente-Sorolla E.; Sanz-Ortega L.; da Silva G. B.; Costo R.; Perez-Yague S.; Talelli M.; Marciello M.; Morales M. P.; Barber D. F.; Gutierrez L. Time-Course Assessment of the Aggregation and Metabolization of Magnetic Nanoparticles. Acta Biomater. 2017, 58, 181–195. 10.1016/j.actbio.2017.05.047. 28536061

[ref65] Ruiz A.; Gutierrez L.; Caceres-Velez P. R.; Santos D.; Chaves S. B.; Fascineli M. L.; Garcia M. P.; Azevedo R. B.; Morales M. P. Biotransformation of Magnetic Nanoparticles as a Function of Coating in a Rat Model. Nanoscale 2015, 7, 16321–16329. 10.1039/C5NR03780H. 26381991

[ref66] Mejías R.; Gutiérrez L.; Salas G.; Pérez-Yagüe S.; Zotes T. M.; Lázaro F. J.; Morales M. P.; Barber D. F. Long Term Biotransformation and Toxicity of Dimercaptosuccinic Acid-Coated Magnetic Nanoparticles Support Their Use in Biomedical Applications. J. Controlled Release 2013, 171, 225–233. 10.1016/j.jconrel.2013.07.019. 23906866

[ref67] Gutiérrez L.; Mejías R.; Barber D. F.; Veintemillas-Verdaguer S.; Serna C. J.; Lázaro F. J.; Morales M. P. Ac Magnetic Susceptibility Study of in Vivo Nanoparticle Biodistribution. J. Phys. D: Appl. Phys 2011, 44, 255002 10.1088/0022-3727/44/25/255002.

[ref68] Gutiérrez L.; Morales M. P.; Lázaro F. J. Prospects for Magnetic Nanoparticles in Systemic Administration: Synthesis and Quantitative Detection. Phys. Chem. Chem. Phys. 2014, 16, 4456–4464. 10.1039/C3CP54763A. 24468801

[ref69] Sun S.; Zeng H.; Robinson D. B.; Raoux S.; Rice P. M.; Wang S. X.; Li G. Monodisperse Mfe_2_o_4_ (M = Fe, Co, Mn) Nanoparticles. J. Am. Chem. Soc. 2004, 126, 273–279. 10.1021/ja0380852. 14709092

[ref70] Moros M.; Pelaz B.; Lopez-Larrubia P.; Garcia-Martin M. L.; Grazu V.; de la Fuente J. M. Engineering Biofunctional Magnetic Nanoparticles for Biotechnological Applications. Nanoscale 2010, 2, 1746–1755. 10.1039/c0nr00104j. 20676420

