# Supplementary material for: Critical Parameters to Improve Pancreatic Cancer Treatment Using Magnetic Hyperthermia: Field Conditions, Immune Response, and Particle Biodistribution
Source: ACS Appl Mater Interfaces. 2021 Mar 12;13(11):12982–96. doi: 10.1021/acsami.1c02338 (PMC8892434; doi:10.1021/acsami.1c02338)
Supplement: Supplementary file 1 — am1c02338_si_001.pdf [file am1c02338_si_001.pdf]

Supporting information

Critical Parameters to Improve Pancreatic Cancer Treatment Using Magnetic Hyperthermia: Field Conditions, Immune Response and Particle Biodistribution

*Lilianne Beola,[±, #] Valeria Grazú,[±,≠, *] Yilian Fernández-Afonso,[±, #] Raluca M. Fratila, [±,≠] Marcelo de las Heras,[¥] Jesús M. de la Fuente, [±,≠] Lucía Gutiérrez[±,≠, #,*] Laura Asín [±,≠,*]*

[±] Instituto de Nanociencia y Materiales de Aragón (INMA), CSIC-Universidad de Zaragoza, 50018 Zaragoza, Spain.

[#]Department of Analytical Chemistry, Universidad de Zaragoza, 50018 Zaragoza, Spain.

[≠]Centro de Investigación Biomédica en Red de Bioingeniería, Biomateriales y Nanomedicina (CIBER-BBN), 50018 ZaragozaSpain.

[¥]Department of Animal Pathology, Universidad de Zaragoza, 50009 Zaragoza, Spain.

**V.G. vgrazu@unizar.es;L.A.: lasin@unizar.es; L.G.: lu@unizar.es*

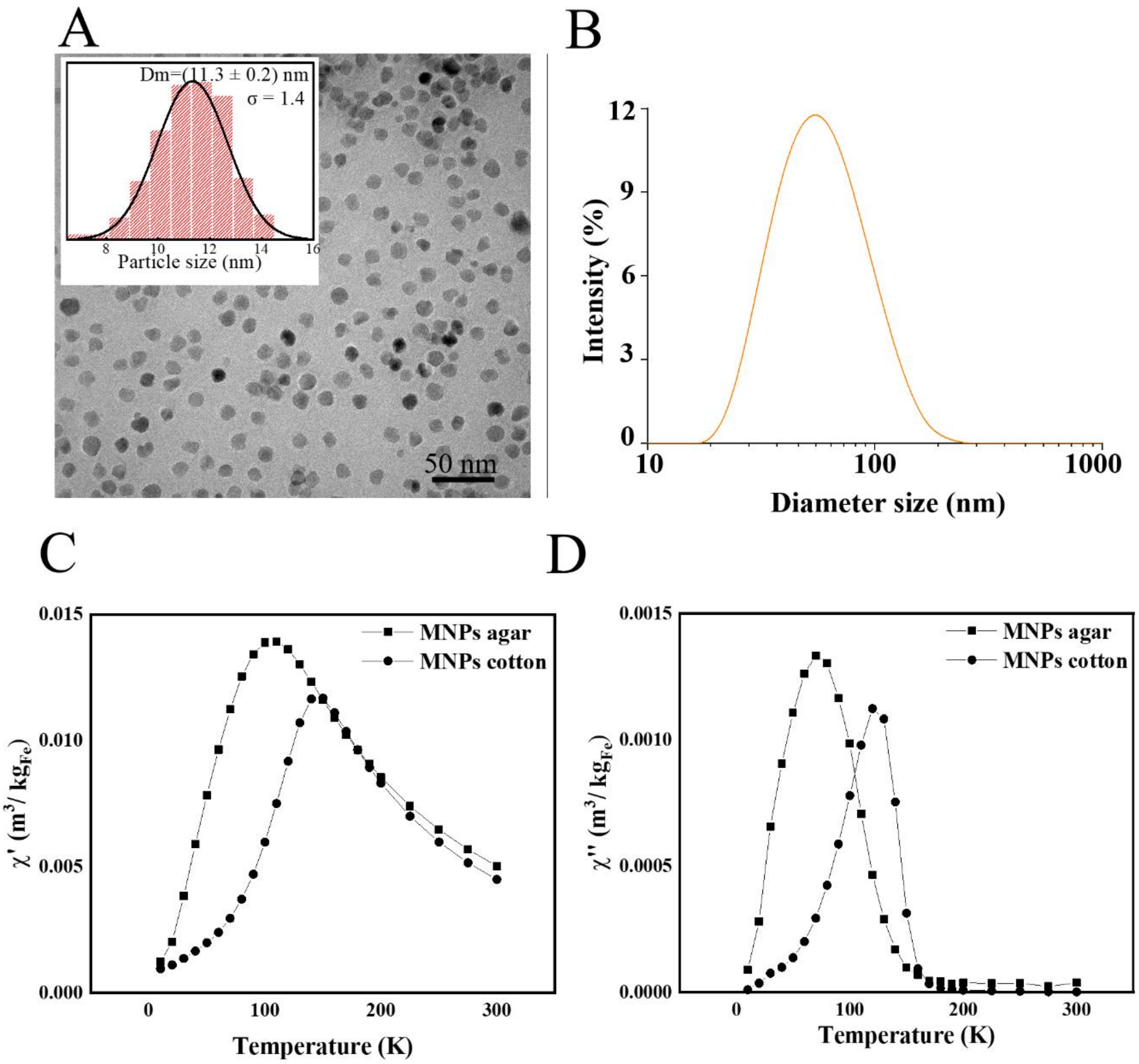

Figure S1. Characterization of the magnetic nanoparticles. (A) Transmission electron microscopy image and nanoparticles size distribution analysis. (B) Hydrodynamic size of MNPs functionalized with glucose in PBS. (C) and (D) Temperature dependence of the AC magnetic susceptibility (in phase and out-of phase components) of the particles prepared in two different ways (dispersed in agar or deposited in cotton) to generate different degree of dipolar interactions.

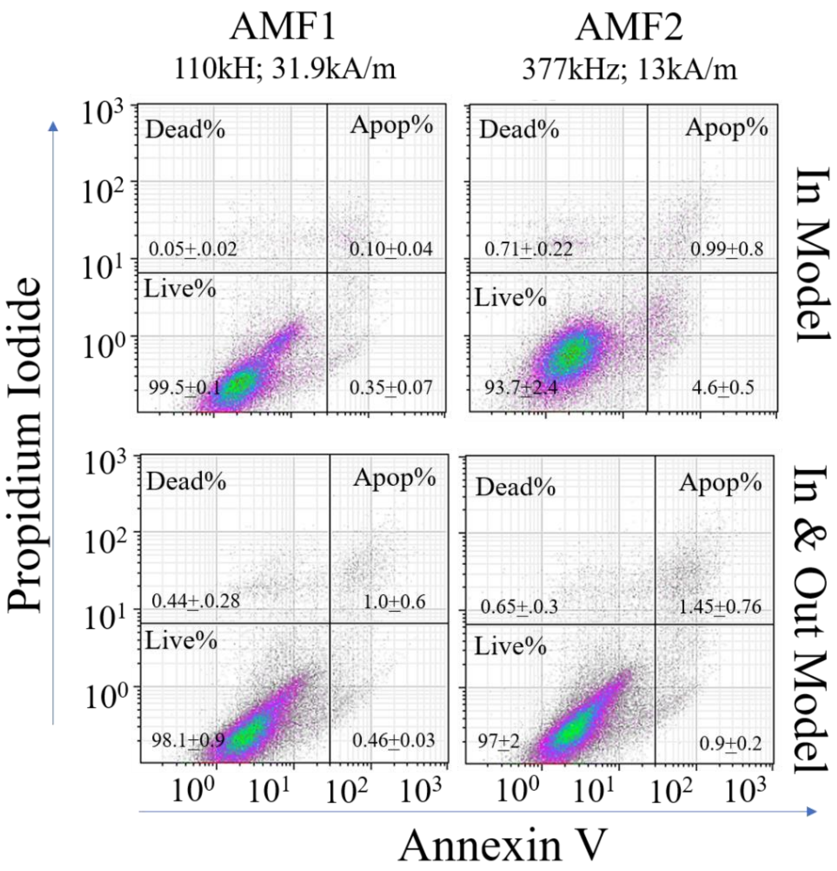

Figure S2. Cell death induction (Annexin V/PI staining) 24 h after AMF exposure of 3D cell cultures of cells, not loaded with MNPs. AMF 1: 110 kHz; 31.9 kA/m. AMF 2: 377 kHz; (Above) In Model and (Below) In &Out Model. Selected density plots were representative of three independent experiments.

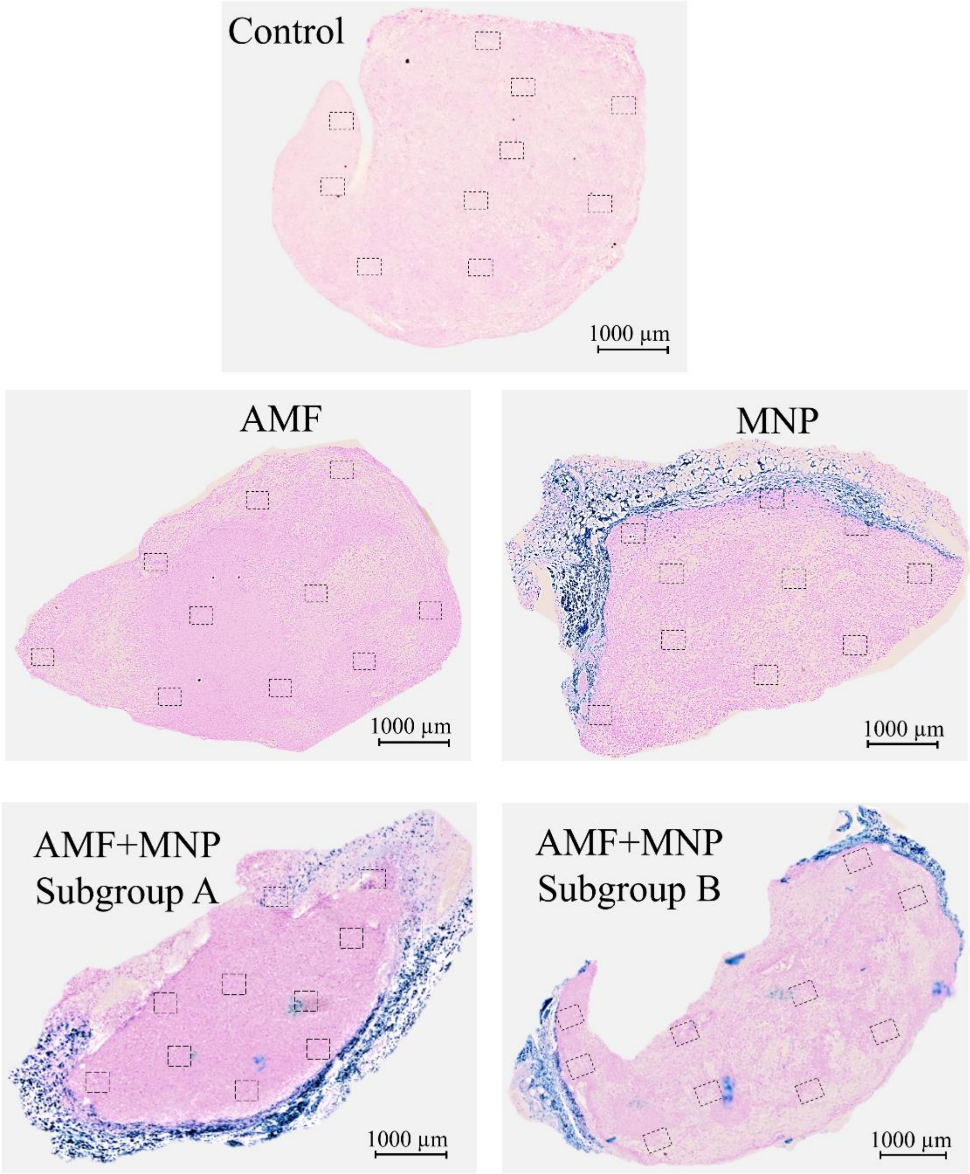

Figure S3. Histological assessment of tumor sections after Perls Prussian blue staining. The squares marked in each image represent ten random areas used for the dead cell quantification in each section.

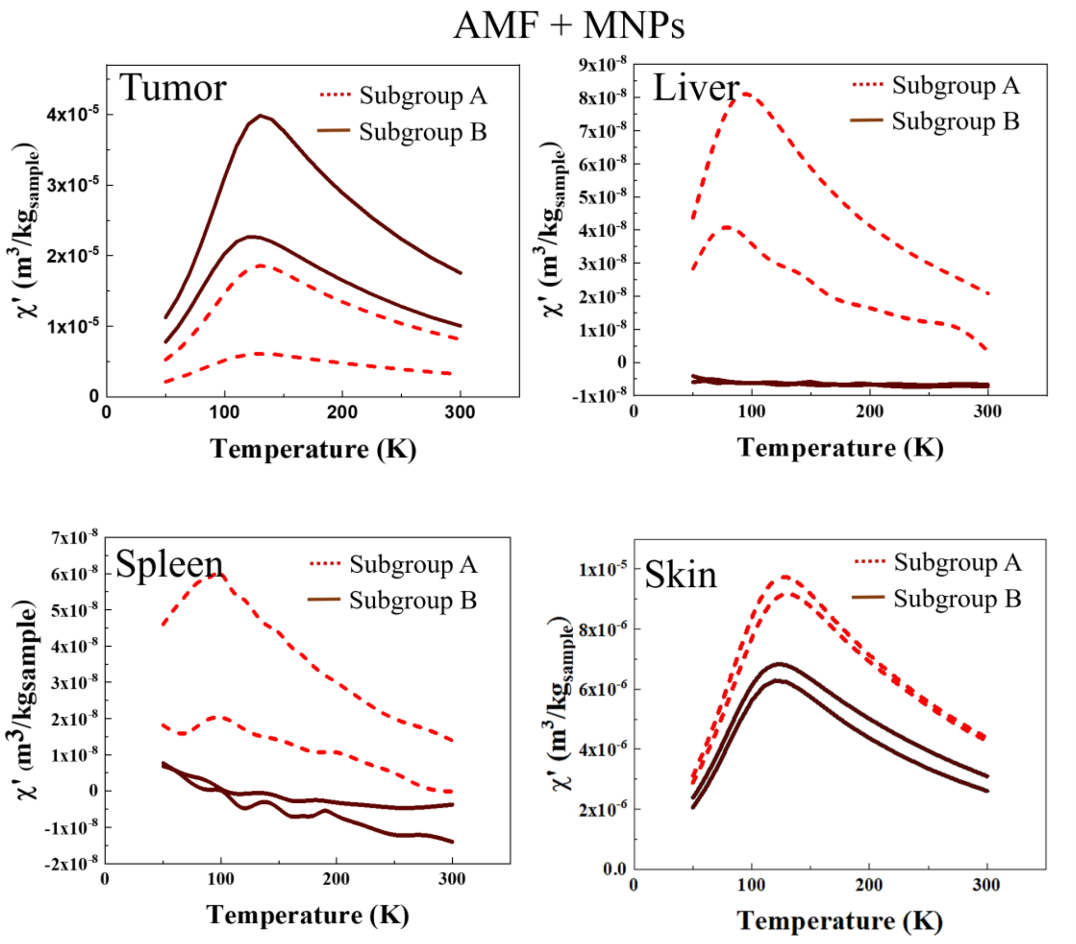

Figure S4. Magnetic characterization of tissue samples. Temperature dependence of the in-phase magnetic susceptibility of tumor, liver, skin and spleen tissues obtained four weeks after the intratumor MNP administration.
